# The Use of Heptamethine Cyanine Dyes as Drug-Conjugate Systems in the Treatment of Primary and Metastatic Brain Tumors

**DOI:** 10.3389/fonc.2021.654921

**Published:** 2021-06-01

**Authors:** Elizabeth Cooper, Peter J. Choi, William A. Denny, Jiney Jose, Mike Dragunow, Thomas I.-H. Park

**Affiliations:** ^1^ Department of Pharmacology, Faculty of Medical and Health Sciences, University of Auckland, Auckland, New Zealand; ^2^ Auckland Cancer Society Research Centre, School of Medical Sciences, University of Auckland, Auckland, New Zealand; ^3^ Neurosurgical Research Unit, The Centre for Brain Research, University of Auckland, Auckland, New Zealand; ^4^ Hugh Green Biobank, The Centre for Brain Research, University of Auckland, Auckland, New Zealand

**Keywords:** heptamethine cyanine dyes, conjugation, drug delivery, brain metastases, primary brain tumor, chemotherapy

## Abstract

Effective cancer therapeutics for brain tumors must be able to cross the blood-brain barrier (BBB) to reach the tumor in adequate quantities and overcome the resistance conferred by the local tumor microenvironment. Clinically approved chemotherapeutic agents have been investigated for brain neoplasms, but despite their effectiveness in peripheral cancers, failed to show therapeutic success in brain tumors. This is largely due to their poor bioavailability and specificity towards brain tumors. A targeted delivery system might improve the efficacy of the candidate compounds by increasing the retention time in the tumor tissue, and minimizing the numerous side effects associated with the non-specific distribution of the chemotherapy agent. Heptamethine cyanine dyes (HMCDs) are a class of near-infrared fluorescence (NIRF) compounds that have recently emerged as promising agents for drug delivery. Initially explored for their use in imaging and monitoring neoplasms, their tumor-targeting properties have recently been investigated for their use as drug carrier systems. This review will explore the recent developments in the tumour-targeting properties of a specific group of NIRF cyanine dyes and the preclinical evidence for their potential as drug-delivery systems in the treatment of primary and metastatic brain tumors.

## Highlights

- Keywords used to search for the literature used in this review pertaining to the field of heptamethine cyanine dyes.
- Heptamethine cyanine dyes, MHI-148, IR-783, IR-780, DZ-1, IR-786, drug-dye conjugate, heptamethine carbocyanine dyes, *near infrared dye, cyanine conjugate.
- Truncations were used in the terms as shown to ensure thorough capture of research pertaining to the field. Literature pertaining to the field of HMCDs in the use of photodynamic therapy were generally excluded, unless the dyes were conjugated to a small molecule inhibitor, as the application of HMCDs in photodynamic therapy has been thoroughly reviewed.

## Introduction

### Brain Tumors and Metastases

The term ‘brain tumors’ refers to a mixed group of neoplasms growing inside the central nervous system (CNS) (1). Primary brain tumors, such as glial tumors and medulloblastomas, can be distinguished from brain metastases originating from a tumor located outside of the brain. Each type of tumor possesses its own biology, treatment, and prognosis, but the functional neurological consequences are frequently similar.

In adults, the global age-standardized rate of primary brain tumors is approximately 6.8 per 100,000, with the highest rates seen in Europe (2). Of the primary brain tumors, astrocytomas are the most common and most fatal, accounting for more than half of all primary brain tumor deaths. Distinguished by histopathological criteria and genetic alterations, astrocytomas can be graded; from the lower grade pilocytic astrocytomas (Grade I), to low grade astrocytomas and oligodendrogliomas (grade II), anaplastic astrocytomas and oligodendrogliomas (grade III), and glioblastomas (grade IV). The prognosis for high-grade astrocytomas are poor, with a median survival of 12-14 months for glioblastoma (GBM) and 24 months for anaplastic astrocytoma (3).

The only established treatment for high-grade astrocytomas consists of maximal surgical resection of the tumor, followed by concurrent radiation and chemotherapy with temozolomide (TMZ), prolonging the median survival by only four months (3). Several chemotherapies are available for second-line treatment, but no standard of care has been established (4–6). Nitrosoureas are blood-brain barrier (BBB)-permeable DNA alkylating agents and include carmustine, lomustine, nimustine and fotemustine (4). Although nitrosourea agents have shown similar efficacy to TMZ, they are associated with substantial hematologic and long-lasting hepatic and pulmonary toxicities, preventing the administration of additional treatments (4). Due to the current limitations of small molecule anti-neoplastic agents, antibody-based therapies have been investigated for the treatment of brain tumors [reviewed in (7)]. Bevacizumab, sold under the brand name Avastin, is a humanized monoclonal antibody against the vascular-endothelial growth factor-A ligand that inhibits angiogenesis (8). Following the results from successful phase III clinical trials in patients with advanced metastatic cancers [reviewed in (9)], bevacizumab was granted full accelerated approval for the treatment of recurrent glioblastoma that had progressed following prior therapy. However, recent clinical trials have shown conflicting results, as favorable clinical effects have not translated into an overall survival benefit (5, 10). The effects of bevacizumab appear transient, with most patients progressing after 3-5 months (6, 11).

In children, brain tumors represent the second most common cancer, comprising approximately 24% of all pediatric malignancies (12). Medulloblastoma is among the most common malignant childhood brain tumor, usually diagnosed between 6-8 years of age (13). Despite a rigorous trimodal therapy regimen, including surgical resection, chemotherapy and cranio-spinal radiation, less than 70% of patients survive beyond five years (13). Moreover, the severe neurocognitive, neuroendocrine and psychosocial deficits attributed to the standard of care underpin the motivation to improve therapeutic strategies in the treatment of medulloblastoma, and other pediatric brain cancers (13).

Metastatic tumors are the most frequent type of intracranial tumor in adults, with reported incidence rates between 2-14 persons per 100,000 population (14). Patients with brain metastasis have not benefited from the recent advances in targeted chemotherapies used in the treatment of the primary tumors, due largely to a lack of BBB penetration. With a less expansive growth pattern than gliomas, tumor cells in metastatic lesions are more protected from systemic chemotherapies by the BBB (14). That is, brain metastases tend to grow in confined colonies, where the BBB remains relatively intact (15). Consequently, there is a higher incidence of the brain becoming the first site of relapse for metastases in patients treated with chemotherapy (14).

### The Blood-Brain Barrier—A Significant Challenge

Chemotherapeutic treatments for primary and metastatic brain tumors are limited by the reduced BBB penetration of the existing anticancer agents (16). The BBB separates the brain from the systemic circulation, controlling the movement of molecules across the vessel walls (16). With the exception of the few drugs approved for the treatment of brain tumors (e.g. TMZ), anticancer drugs are generally excluded from the brain by this barrier (3, 5). Tumors are also known to compromise the integrity of the BBB, resulting in a heterogeneous vasculature known as the blood-tumor barrier (16). The blood-tumor barrier is characterized by numerous distinct features, including non-uniform delivery and active efflux of molecules – all of which further complicates the delivery of anticancer agents (16). An improved delivery of potent anticancer drugs into the brain is, therefore, an ongoing challenge in the chemotherapeutic treatment of brain tumors.

The BBB is comprised of a functionally complex, tightly regulated neurovascular unit that includes endothelial cells, pericytes and astrocytic end feet (16). Together, these cells control the movement of cells and molecules to maintain the homeostatic neuroparenchyma environment. However, these mechanisms also interfere with the delivery of approved anticancer therapeutics into the brain to target brain tumors (16–18). Endothelial cells of the CNS have polarized cellular transporters which regulate influx and efflux between the neuroparenchyma and blood (16). ATP-binding cassette (ABC) transporters are expressed on the luminal and abluminal sides of the vessel walls and mediate the efflux of xenobiotics and toxins away from the neuroparenchyma space (19). Unfortunately, most low molecular weight anticancer agents are substrates for ABC proteins (16–18). These ABC transporters, such as the breast cancer resistant protein and the multi-drug resistance-associated proteins, are highly expressed in the capillary endothelial cells constituting the BBB (19). Another important efflux protein present in the BBB is the P-glycoprotein (19). P-glycoprotein can efflux small molecule tyrosine kinase inhibitors (TKIs) used as chemotherapy agents, such as dasatinib, imatinib, vandetanib and nintendanib (17), as well as the standard GBM chemotherapy agent, TMZ (18). Therefore, provisioning strategies to circumvent or nullify these transporters are essential in developing agents to target brain tumors.

### Overcoming the Blood-Brain Barrier

A number of strategies have been investigated to overcome the limitations of the BBB in the treatment of brain cancers: the first is to bypass the BBB, the second is to increase its permeability, and the third is to use carrier molecules that are permeable to the BBB (20–22).

#### Bypassing the Blood-Brain Barrier: Intraventricular Infusion and Intracerebral Implants

Intraventricular infusion and intracerebral implants are the main strategies used to bypass the BBB (21, 23–25). Intraventricular infusions, however, have been unsuccessful due to the relatively small surface area of the cerebral spinal fluid, limiting the diffusion of anticancer drugs into the brain parenchyma (17). In addition, intracerebral implants of wafers impregnated with anticancer agents can be placed into the tumor cavity during surgery. These have shown survival benefits in patients with newly diagnosed malignant glioma, albeit, only lasting several weeks (20). The efficacy of both strategies was limited by the diffusion capacity of the drugs (20). In contrast, the cerebral vasculature has an extensive network that, on average, is only separated by 40 µM, and accounts for almost 15% of the cardiac output under resting conditions (26, 27). The cerebral circulation, therefore, would be the ideal route to achieve drug delivery.

Tumor Treating Fields (TTFields), developed by Novocure, is a non-invasive, loco-regional anti-mitotic treatment which uses alternating electric fields in the intermediate frequency range (200 kHz) to disrupt cancer cell division with minimal systemic toxicity (28). TTFields are administered to patients using a patient-operated home-use Optune device, delivering alternating electric fields to arrays affixed to the scalp (28). The phase III clinical trial in newly diagnosed GBM demonstrated a significant increase in overall survival by 4.9 months when combined with TMZ, in comparison to patients receiving TMZ alone (20.9 months and 16.0 months, respectively), resulting in the FDA approval of TTFields for GBM in 2015 (29).

#### Increasing the Permeability of the Blood-Brain Barrier

To overcome the limitations of the BBB, several approaches to physically and chemically disrupt the barrier have been developed. The main strategies include osmotic disruption through intracarotid infusions of mannitol, modification of tight junctions by the administration of bradykinin analogues, and focused ultrasound techniques (21, 24, 25). The superselective intra-arterial delivery of mannitol prior to the infusion of chemotherapy has been shown to disrupt the BBB and increase drug delivery by opening endothelial cell gap-junctions (24). Analogues of endogenous peptides, such as bradykinin have also been explored to increase the permeability of the BBB by activating cerebral vascular receptors (21). Although these strategies have been shown to increase the delivery of chemotherapies into the brain, in comparison to systemic injection, the effect is transient, with the delivery reversed within minutes (21). In addition, opening of the BBB with low-intensity pulsed ultrasound has emerged in the last two decades as a technique to enhance drug delivery into the brain (30). Beccaria et al. reviewed the preclinical data of several focused ultrasound techniques and highlighted the clinical trials currently underway (30). These strategies, however, are hindered by neurological toxicities associated with the lack of specificity in what substrates can cross the disrupted BBB and show conflicting survival benefits in clinical trials (31).

#### Carrier Molecules to Overcome the Blood-Brain Barrier: Nanoparticle Formulations

Nanoparticles have been explored to protect drug entities in the systemic circulation and facilitate the uniform delivery of drugs across previously impenetrable barriers (9). Several clinical trials have focused on encapsulating and delivering chemotherapeutics in nanoparticles for the treatment of brain tumors and metastases (9). The delivery of nanoparticles into brain tumors, however, has been largely unsuccessful (32). A principal challenge of nanoparticle delivery is their biodistribution, where many of the approved nanoparticles are polyethylene glycol (PEG)γ-lated or PEG terminated, which promotes clearance by immune cells (32). Moreover, the scale-up and reproducibility of nanoparticle formulations have been onerous (33). The success of a drug-delivery system relies on the synthesis of an inert, consistent and reproducible product, and although promising, nanoparticles are yet to deliver a formulation that accumulates in brain tumors at an effective concentration [reviewed by (9, 33)]. Drug-delivery systems, therefore, hold great potential for improving the bioavailability and tumor-specificity of anticancer agents, with promising discoveries emerging at a rapid pace.

### Chemotherapy-Induced Neurotoxicity: A Lack of Specificity

Traditional and modern chemotherapy and radiotherapy can have undesirable effects on the CNS, which can result in the discontinuation of the treatment or dose-adjustments that prevent the achievement of a desirable therapeutic outcome [reviewed by (34)]. Traditional radiation and chemotherapy act on dividing cells by inducing DNA damage or inhibiting DNA repair. Albeit slowly, the resident population of stem cells that replenish neuronal and glial populations undergo collateral damage (35). There is evolving evidence to suggest that cognitive decline following cancer treatments could be a consequence of impaired hippocampal and subventricular zone neurogenesis. Rodent studies demonstrate that post-radiation, the subventricular zone neurogenesis undergoes a delayed recovery, while hippocampal neurogenesis remains stalled (36). Immunohistochemical analysis of post-mortem human brain tissue revealed that pediatric and adult medulloblastoma patients treated with surgery, radiation and chemotherapy had extensive ablation of hippocampal neurogenesis compared to controls, reinforcing findings from experimental models (37, 38). However, there is a recent study stating that adult hippocampal neurogenesis might be limited, and hence these effects could be exaggerated in rodent models (39). Of note, reports suggest that radiation and chemotherapy not only affect the stem cell pool, but also alter the neurogenic environment [reviewed in (40)]. Specifically, radiation-induced activation of microglia and subsequent elaboration of pro-inflammatory cytokines directly impair neuronal differentiation (41). Radiation and chemotherapy treatment are also reported to cause indirect damage to neural structures through vascular damage, fibrosis, and disruption of endocrine signalling (41). Given the slow rate of cell turnover in the CNS, and the evolving indirect toxicities, symptoms of neurological deficits can be delayed by order of weeks to years following cessation of therapy (35).

Radiation necrosis is a delayed complication of radiation therapy for brain tumors and a dose-limiting factor for stereotactic radiotherapy, a standard treatment for brain metastases (42, 43). Generally occurring months to years after radiation treatment, radiation necrosis is thought to be a consequence of a combination of vascular injury, glial and white matter damage (44). Consequently, radiation necrosis is often associated with cognitive dysfunction, seizures, or focal neurological deficits that affect the patient’s quality of life (43). In addition, the radiological differentiation of tumor recurrence and necrosis is challenging (43).

Acute and chronic complications of the CNS following chemotherapy treatment are common. Acute complications include headaches, seizures and acute encephalopathy, which can have detrimental effects on patient outcome, limiting the duration of treatment (34). Chronic encephalopathy can result in mental impairment and structural changes that develop months to years after receiving CNS-directed chemotherapies (34). These can have detrimental effects on patient outcome, including severe weakness, dementia and even death. In addition to the overt cognitive deficits, many patients experience a more subtle syndrome of cognitive dysfunction following high-dose chemotherapy treatments, which is commonly referred to as “chemobrain” (34). Chemotherapy side effects may also occur in the peripheral nervous system (45). Several neuroprotective strategies have been explored to prevent these conditions, but clinical trials examining their effectiveness have been negative (46, 47). As brain cancer treatments continue to develop, minimizing off-target toxicities is paramount to ensure that patients can proceed with their full treatment course without experiencing further neurological injuries. To achieve this, various drug delivery systems that exploit tumor-intrinsic properties have been explored to deliver drug payloads to the tumor with high specificity (48, 49). Heptamethine cyanine dyes (HMCDs) have recently emerged as a potential drug delivery system that can overcome the challenges surrounding BBB penetration and tumor specificity.

## Tumor-specific Heptamethine Cyanine Dyes

**Figure 1:**
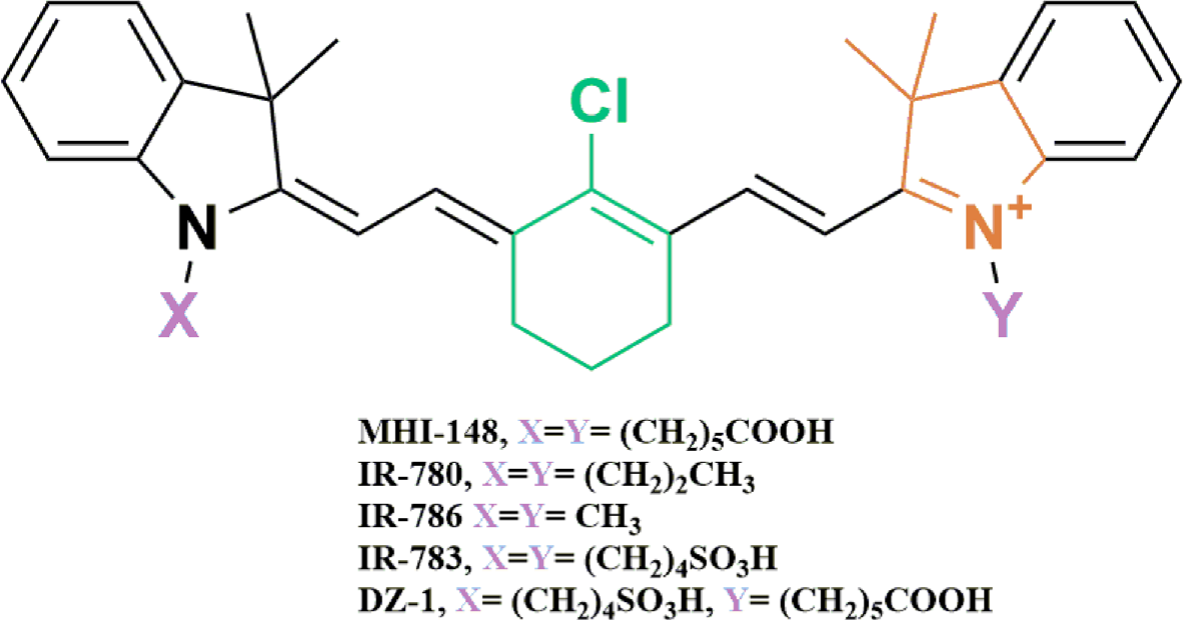
Chemical structures of near-infrared heptamethine cyanine dyes used as tumor-specific drug-carrier agents. The presence of the fused cyclic meso-Cl cyclohexene ring (Green), and the ionizable functional group (Purple) attached to the terminal tetramethyl indole ring (Orange), is thought to contribute to the tumor selectivity of HMCDs.

Existing research recognizes the preferential tumor uptake of a class of NIRF HMCDs. This has been demonstrated in a variety of cancer cell lines, tumor xenografts, spontaneous mouse tumors in transgenic animals and human tumor samples (references in **Table 1**). Originally identified for their fluorescent and mitochondrial-targeting tumor-imaging properties, they have recently been explored as drug carriers to deliver chemotherapeutics to tumors (78, 79). HMCDs can be utilized as drug-delivery systems by attaching non-selective drugs to the peripheral carboxylic acid groups to make amides and esters, or by nucleophilic substitution of the meso-Cl group on the core cyclohexenyl skeleton (**Figure 1**). The tumor-specificity, retention and BBB-permeability properties of this group of NIRF agents provides a unique platform for their use as a small-molecule drug-carrier system.

**Table 1:** Summary of HMCD studies on human tumor cell lines and xenografts.

Tumor Type	Tumor Cell-Line	Heptamethine Cyanine Dye	Conjugate	References
Lung Cancer	A549, NCIH-460, H358, A549-DR	IR-783, IR-780, MHI-148	Methotrexate, Erlotinib	(50–57)
Breast Cancer	MCF-7, MDA231, LTED	IR-783, IR-780, MHI-148, DZ-1	Methotrexate, FTS, Genistein, Erlotinib	(51, 55–61)
Hepatoma	SMMC-7721, HepG2	IR-783, IR-780, MHI-148	Dasatinib, Methotrexate	(51, 53, 55, 62)
Cervical Cancer	HeLa	IR-783, IR-780, MHI-148		(51, 53, 60)
Prostate Cancer	PC-3, LNCap, C4-2, DU-145	IR-783, IR-780, IR786, MHI-148	MAOA inhibitor, Gemcitabine, Isoniazid	(53, 63–68)
Leukemia	K562	IR-783, MHI-148		(53, 63)
Pancreatic Cancer	MIA, PaCa-2	IR-783		(53)
Renal Cancer	SN12C, ACHN, Caki-1	IR-783, IR-780		(69)
Glioblastoma	U251, GL261, U87, T98T, LN18, Primary PDX	IR-780, IR783, DZ-1, IR-786	Gemcitabine, Crizotinib, Dasatinib, MAOA inhibitor, Rucaparib	(54, 70–76)
Osteosarcoma	MG-63	IR-780		(77)

Several NIRF dyes have become commercially available, including rhodamine, BODIPY for use in the detection and monitoring of neoplasms in animal models (80). Indocyanine green (ICG) and methylene blue however, are the only NIRF agents approved by the FDA for use in medical diagnostics (80, 81). Other red-flourescent dyes, such as 5-ALA, are also used in fluorescent-guided surgery for the resection of high grade gliomas (82). The use of 5-ALA in fluorescent-guided surgery is challenged by the strong absorption of the excitation light by red-blood cells, making the underlying tumor fluorescence difficult to visualize, in addition to the lack of absorption of 5-ALA by lower grade tumors (83). The NIRF and tumor-specific accumulation of HMCDs may also potentiate their use in fluorescent-guided surgery, and tumor monitoring (78, 84, 85). A deeper understanding of the photophysical properties, stability, and *in vivo* targeting capabilities is needed for their development as dual targeting and imaging therapeutics.

HMCDs exhibit key structural features that are thought to render them tumor selective. The first is the presence of a fused cyclic chloro-cyclohexene ring (green), second, they possess an alkyl chain that attaches to the terminal tetramethyl indoline ring (orange), and they contain an ionizable functional group, such as carboxylic or sulfonic acid (purple) (**Figure 1**) (70, 86). Specific chain lengths also contribute to the observed selectivity of dyes towards tumor tissues. That is, dyes with a great number of carbons in the side chain were reported to alter the hydrophobicity and rigidity of the dyes, hence affecting their uptake into cancer cells (86).

Recently, a group of HMCDs that preferentially target and accumulate in tumors have been described (IR-780, IR-783, IR-786, MHI-148 and DZ-1; references in **Table 1**). These dyes are reported to preferentially accumulate in the mitochondria and lysosomes of tumor cells, but not normal cells (84, 85). In a preclinical trial, MHI-148 was applied in a surgically removed kidney from a patient with renal cell carcinoma (63). *Ex vivo* NIRF imaging demonstrated that fluorescence signal intensities were six-fold higher in the tumor when compared to the normal surrounding kidney tissues. These studies highlight the potential clinical translation of these NIRF dyes as tumor-targeting anti-neoplastic agents.

Tumor-specific HMCDs, however, are not devoid of biological activity. Several groups have demonstrated that HMCDs alone have some cytotoxic activities at higher supra-micromolar concentrations. Yi *et al.* demonstrated significant concentration-dependent cytotoxic and anti-migratory effects of IR-780 on prostate cancer cell lines (50, 64). Similarly, our group has shown that IR-786 was cytotoxic to patient-derived GBM cells, with an EC_50_ of 1.7 μM. It also exhibited cytotoxic synergism with TMZ, as co-treatment reduced the EC_50_ by four-fold to 400 nM (71). Moderate toxicity has also been reported with IR-780 on the murine breast cancer 4T1 cell line (less than 30% reduction in cell viability with up to 16 μM), and IR-783 on the human MCF-7 breast cancer cell line (EC_50_ of 25 μM) (58, 87). Although cytotoxic activity is evident at supra-micromolar concentrations, further studies are required to understand the structure-activity relationships across HMCDs. Evaluating the toxicity of HMCDs across multiple tumor and non-tumor cells lines will allow researchers to identify which structures are biologically inert, and thus more favorable as a drug-delivery system.

### Expanding the Indications of Existing Chemotherapies for the Treatment of Brain Cancer

Advances in our understanding of tumorigenic pathways have allowed existing anticancer agents to be successfully repurposed to treat other cancers that share common signaling pathways (17). However, the expansion of existing anticancer agents for the treatment of brain tumors and metastases has been challenging. Dasatinib, for example, was originally identified as a dual inhibitor of BCR/ABL gene-encoded tyrosine kinase and the Src family of tyrosine kinases. It was subsequently granted accelerated approval for the treatment of chronic myeloid leukemia but was found to inhibit the platelet-derived growth factor receptor (PDGFR) alpha and beta with nanomolar potencies (88). Preclinical and *in vitro* studies support an important role of Src and PDGFR in human glioblastoma. However, *in vitro* and *in vivo* studies have revealed that dasatinib is a substrate for efflux transporters that are expressed on the BBB (89). This likely explains the lack of success in clinical trials exploring dasatinib in the treatment of primary brain tumors (90, 91). Hence, carrier molecules like HMCDs may provide a platform to repurpose drugs, like dasatinib, for the treatment of brain tumors which have been previously excluded from the brain (**Table 1**).

### The Mechanism of Uptake of HMCDs

HMCDs and their published conjugates have been shown to persist in tumor tissue *in vivo* over periods of several days, despite the half-lives of these compounds in serum being on the order of minutes to a few hours (49, 70, 72, 73). Most research suggests that preferential tumor uptake is mediated by organic anion-transporting polypeptides (OATPs), but recent evidence also suggests a clear role for albumin and the use of endocytosis mechanisms in the uptake and persistence of these tumor-specific dyes (62, 70, 71).

#### The Role of OATPs in the Uptake of HMCDs

This assertion for the involvement of OATPs is reasonable as hypoxia triggers the activation of HIF1-alpha (HIF1α), which promotes OATP expression (70). OATPs are promiscuous transporters; they influx organic anions, including bile salts, steroids, bilirubin and thyroid hormones, and can take up unnatural organic molecules (92). Bicarbonate is excreted to balance the charge; hence, molecules imported *via* OATPs are not pumped out in the same manner (92). OATPs have also been shown to be overexpressed by a number of different cancers and on the endothelial cells of the BBB; therefore, manifest as a logical platform for achieving BBB and blood-tumor barrier permeability (93).

The rationale for the involvement of OATPs in the uptake of these NIRF agents was formulated on a simple experimental *in vitro* paradigm that compared the fluorescent intensity of cells in serum-free culture media. Typically, cells treated with a pan-OATP inhibitor demonstrated a reduction in fluorescent intensity, implying that the uptake of the NIRF agents were, in part, *via* the OATPs. In contrast, cells treated with hypoxia-mimetic agents, such as dimethyloxalylglycine and cobalt chloride, stabilized HIF1α and increased the expression of OATPs, thus resulting in an increase in the fluorescent intensity. Several groups have reported that upon uptake, HMCDs are localized to the mitochondria of tumor-cells (53, 56, 60, 67). Interestingly, a recent study reported that the mitochondrial uptake of the IR-780 dye was mediated by the mitochondrial inner membrane transporter ATP-binding cassette sub-family B member 10 (ABCB10), and a siRNA-mediated knock-down of the ABCB10 transporter transcript in cancer stem cells reduced the uptake of IR-780 significantly (94). Although ABCB10 is not an OATP, there is a clear relationship between HIF1α, and the uptake of IR-780 into tumor cells. Notably, HIF1α was bound directly to the ABCB10 gene promoter region, and HIF1α expression correlated with the expression of ABCB10. Therefore, there is a clear signaling axis between OATPs and HIF1α that plays a role in the uptake of HMCDs into tumor cells. However, it has been observed that the meso-Cl group on the HMCD core structure is displaced by S-nucleophiles under physiological conditions, including the free cysteine residue in serum albumin and glutathione (74, 95, 96). It is important to note that the inhibition of OATPs failed to completely inhibit the uptake of the dye into tumor cells (60, 71). Therefore, it is likely that other mechanisms for transporting HMCDs into the tumor exist (96).

#### The Role of Albumin in the Uptake of HMCDs

Albumin was reported to naturally accumulate in several types of solid tumors, including sarcomas, lung cancers, and GBM, where it is used as a primary nutrient source by cancerous cells (97, 98). Due to the active consumption of nutrients, cancer cells have been shown to overexpress nutrient transporters to meet their increased demand for energy. For example, albumin-binding proteins are highly expressed in malignant cells and tumor vessel endothelial cells responsible for albumin uptake. Additionally, albumin has been shown to accumulate in high quantities within brain tumors due to the enhanced permeability and retention (EPR) effect (99). The EPR effect is a consequence of the development of abnormal vasculature within the growing tumor with functional and anatomical abnormalities. This results in extensive leakage of blood plasma components, such as albumin, into the tumor interstitium (97–99). Additionally, poor lymphatic clearance and a slow venous return mean that macromolecules are retained in the tumor tissue. Utilizing the EPR effect is a strategy explored to deliver anticancer drugs selectively to the tumor (99). Traditional low molecular-weight anticancer agents lack tumor selectivity, resulting in wide distribution to healthy tissues and organs and severe systemic toxicity (99). Therefore, the ability of a drug to interact with albumin would likely result in enhanced tumor tissue retention and reduced clearance.

Several groups have successfully explored albumin-based drug delivery using Cys_34_ as a chemical conjugation approach, such as Abraxane, the first albumin-based drug approved in oncology (100–102). Furthermore, the association of ICG to albumin is thought to underpin the accumulation in tumor regions with enhanced vascular permeability (103).Albumin has 14 disulphide bonds and one unique, free cysteine residue, Cys_34_ (104). The pKa of the Cys_34_ albumin thiol group is relatively low (~5) in comparison to the pKa of other low molecular weight aminothiols present in the plasma (8.5-8.9 for Cys and GSH, respectively) (105). This means at a physiological pH, Cys_34_ exists as a thiolate anion and is highly reactive with disulphides and thiol (104). Present in high concentrations in the blood, it acts as a carrier for small molecules, many of which non-covalently bind to one of two binding sites, Sudlow sites I and II. Sudlow site I preferentially bind heterocyclic compounds like warfarin, whereas Sudlow site II is reported to bind to aromatic compounds such as ibuprofen. Covalent binding of endogenous and exogenous compounds with albumin also occurs. However, drugs covalently bound to albumin are reported to require a decoupling event that releases their active form to exert their pharmacological activity (104). Drug interaction with human serum albumin (HSA) tends to enhance the bioavailability of a drug and has a significant role in the pharmacokinetic behavior of drug molecules regarding the half-life, efficacy, reducing toxicity and improving drug-targeting [reviewed in (104)]. Hence, albumin association offers an attractive drug-delivery approach for the treatment of tumors.

Interestingly, the idea of conjugating HMCD to albumin for optical imaging has been described in a patent, despite the displacement of the meso-Cl occurring endogenously with albumin (106). The literature suggests that electrophilic small molecules might react with Cys_34_ directly, or possibly associate with one of the binding sites before being relayed to the free thiol. There is evidence for the relay of non-covalently bound HMCDs to a covalent bond (107, 108). This comes in the form of an immediate UVƛ max red-shift (822 nm) for the HMCD MHI-148 when added to HSA, followed by a gradual blue-shift to another ƛ maximum of 786 nm after 1.5 hours *in vitro (* 107). Hence, the instantaneous reaction of HMCDs in serum is to form a non-covalent adduct with albumin. However, an ICG analogue synthesized by Tan and colleagues demonstrated high albumin binding affinity, resulting in an improved tissue accumulation and tumor selectivity through the EPR effect (58). The authors argue that the asymmetric properties of their dye, with a modified water-soluble carboxyl chain and liposoluble ester, are responsible for its ability to interact with and form complexes with albumin. Furthermore, the micropinocytosis inhibitor, amiloride, reportedly inhibited the uptake of the meso-Cl substituted genistein-IR-783 conjugate by 40% (58). Therefore, it is unclear whether the meso-Cl group would be required for the formation of albumin-complexes or the endocytosis of HMCDs into tumor cells.

#### Exploiting Tumor-Intrinsic Properties, a Role for OATPs, Endocytosis and Albumin?

**Figure 2:**
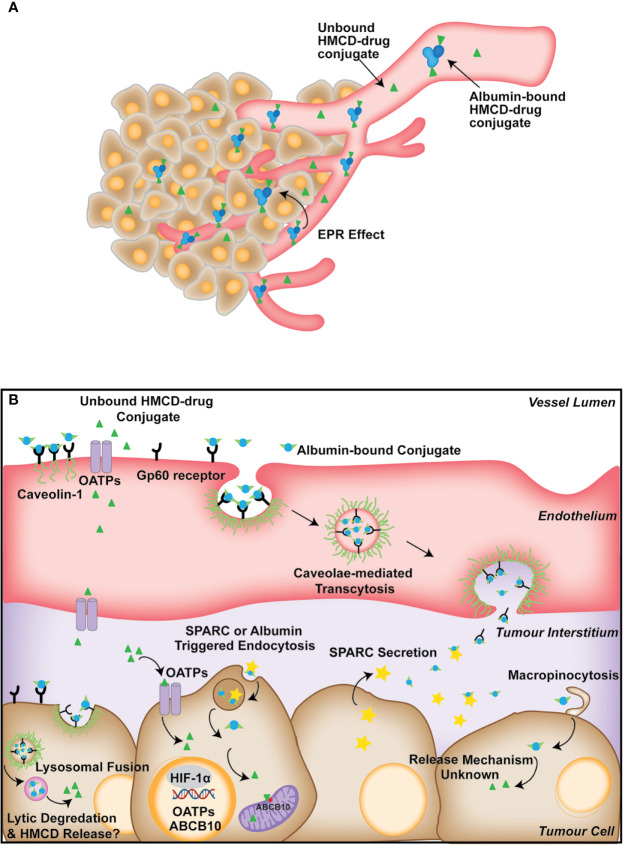
**(A)** Summary of the proposed mechanisms of the tumor-specific accumulation of HMCD-drug derivatives from the circulation. HMCDs travel through the circulation as unbound or albumin-bound molecules toward the bulk tumor through the EPR effect. HMCDs may exist as covalently or non-covalently bound to albumin in the plasma, or as free molecules. **(B)** Describes the proposed mechanism of uptake of HMCD-drug conjugates into tumor cells. Albumin-bound HMCDs bind to caveloin-1 associated Gp60 receptors, triggering caveolae-mediated transcytosis across the endothelium, which is then deposited in the tumor interstitium. The unbound HMCDs are transferred across the endothelium through the polarized expression of OATPs. SPARC is secreted from the tumor cells into the interstitium and entraps albumin and albumin-bound HMCD, which is likely taken up through SPARC or albumin-triggered endocytosis or macropinocytosis. Albumin-bound HMCDs are internalized by the tumor through caveolae-mediated endocytosis, but rather than being transcytosed, caveolae fuse with lysosomes, resulting in lytic degradation of albumin and the subsequent release of the HMCDs. OATP also play a role in the uptake of free HMCD from the tumor interstitium.

By interacting with albumin, the HMCD-carrier can reach the tumor vasculature passively through the EPR effect (109). However, there is a lack of direct evidence surrounding how HMCDs and their conjugates move from the tumor vasculature into the tumor cells. Given the size of the HMCD-drug conjugates, and the reported association with albumin, it is likely that the uptake is mediated by endocytosis mechanisms. Studies have suggested that the uptake of HMCD complexes, including MHI-148-HSA and genistein-IR-783 conjugates, could be mediated by micropinocytosis and lipid-raft endocytosis (**Figure 2**) (58, 74). This is consistent with other macromolecule delivery systems, such as the albumin-paclitaxel conjugate Abraxane, which also utilizes endogenous transport pathways to achieve enhanced tumor tissue distribution (110). In concordance, Abraxane’s tumor uptake was antagonized by inhibition of caveolar-mediated endocytosis, and the knockdown of Cav-1, the structural component of caveolae, also attenuated Abraxane’s anti-tumor properties *in vivo (* 110). This suggests caveolae-mediated endocytosis is critical for the cellular uptake of albumin-associated compounds. Secreted protein acidic and rich in cysteine (SPARC) plays a crucial role in cell growth through its interaction with various cytokines (111). Moreover, SPARC binds to albumin and co-localizes as a bound form in cancer tissues, and its expression is correlated with improved survival in several cancers (111). SPARC has also been shown to mediate the uptake of cyanine dye Cy5-N_3_ in a U87 glioblastoma cell line and xenograft model. This suggests that SPARC could also play a role in tumor uptake of albumin-bound HMCDs, but direct evidence for the uptake of HMCDs is lacking (**Figure 2**) (97). Although SPARC has been investigated for its role in the uptake of albumin-bound molecules such as Abraxane, its involvement in the uptake of HMCDs is yet to be discerned.

### The Intracellular Fate of HMCD-Drug Conjugates

As described, for the HMCD or their conjugates to exert its biological activity, it must be cleaved from the albumin adduct. Studies suggest that the acidic environment of the tumor may facilitate the release of the covalent adducts from albumin through acidic cleavage (108). Tan et al. further demonstrated that pH reduction shifted the NIRF spectra of the albumin-bound HMCD to that of the free dye, suggesting that the acidic environment results in protonation of thiol, which leads to cleavage of the HMCD from albumin. Future studies should further investigate the effects of endocytosis inhibitors on cells cultured in serum-free conditions, devoid of albumin (71, 76). The trafficking and intracellular fate of HMCDs will depend on the mechanism of endocytosis (**Figure 2**). Endocytic pathways rarely operate in isolation, and hence it is important to understand the relative contributions of different endocytic pathways to the activity of the HMCD (112). The persistence and efficacy of HMCDs and their drug-conjugates asserts that HMCDs are not trafficked through a degradative path (71, 108). However, a thorough characterization of the endocytosis of HMCDs will facilitate the design of efficient carrier molecules that are processed by productive endocytic pathways.

The relative contribution of each transport mechanism is unclear, but it is likely that the uptake and persistence of these dyes rely on a combination of the described processes. There is clear evidence to suggest a role for OATP-mediated uptake of unbound HMCD, which we and others have shown *in vitro* in the absence of albumin (71, 74). The endocytosis of HMCDs into tumor cells remains to be thoroughly characterized in serum-free conditions. However, the increased uptake of albumin and the overexpression of its transporters in brain tumor cells is well established, providing a compelling rationale for the advantages of a carrier system that utilizes albumin (109, 111). Indeed, HMCDs could form covalent and non-covalent adducts with albumin, and the uptake of these complexes can be antagonized by inhibitors of endocytosis. Nonetheless, establishing the mechanisms behind the uptake and persistence of HMCDs and their conjugates in tumors will be fundamental to the optimization of HMCDs as efficient drug-delivery systems.

## Investigating the Potential of HMCDs as Drug-Delivery Carriers

### Improving the Therapeutic Potential of Traditional Chemotherapy Agents

#### Gemcitabine

Gemcitabine is a nucleoside analogue currently used for the treatment of various solid tumors. The metabolite of gemcitabine is incorporated into DNA, resulting in chain termination, and cell death by apoptosis (72). Gemcitabine was shown to inhibit DNA chain elongation, act as a potent radiosensitizer, and enhance antitumor immune activity (113). Its use for high-grade brain tumors has been evaluated in clinical trials but has yielded conflicting results. Gemcitabine showed negative results in two phase II clinical trials for the treatment of high-grade gliomas, failing to improve overall survival and progression-free survival (NCT00014170, 1839IL/0116) (17). In addition, gemcitabine has significant drawbacks due to its relatively short half-life (113). It is extensively degraded by cytidine deaminase in the liver, which can result in hematological side-effects due to the compensatory high drug doses and frequent administration schedules (113). In an effort to overcome the lack of bioavailability and specificity, a gemcitabine-HMCD conjugate has recently been evaluated *in vivo* for the treatment of primary and metastatic brain cancers (70, 72). Conjugation with HMCDs could be a logical approach to increase the tumor specificity and bioavailability in brain tumors, whilst reducing the doses required to overcome liver degradation and minimize toxicity.

**Figure 3:**
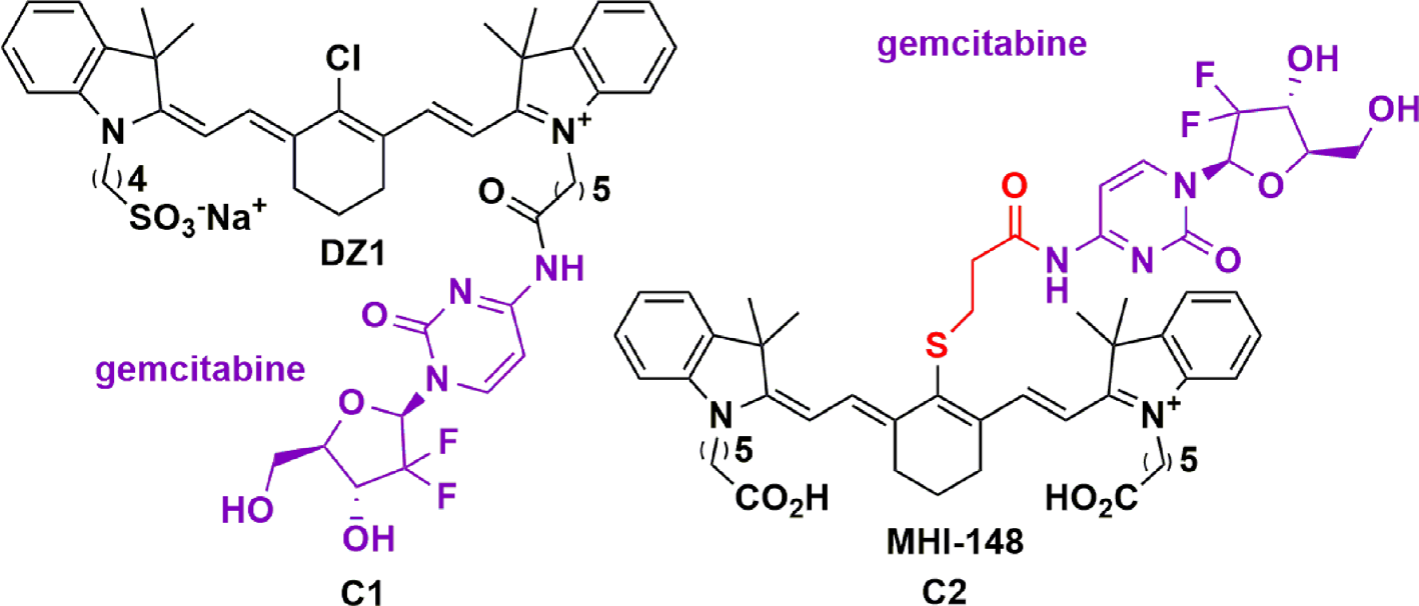
Structure of gemcitabine conjugated to DZI (C1) and MHI-148 (C2) showing tumor specificity in GBM and prostate tumor metastases (70, 72).

HMCDs have been investigated as a carrier for gemcitabine by two groups (70, 72). Wu and colleagues investigated the potential of IR-783 as a drug delivery carrier covalently linked to gemcitabine *via* the carboxylic acid group, leaving the meso-Cl group intact (**Figure 3**, **C1**) **(** 70). Using *in vivo* xenograft models of GBM and prostate tumor metastases, they demonstrated a modest increase in NIRG penetration of the BBB, and hence, tumor bioavailability*. Ex vivo* analysis confirmed an eight-fold increase in the signal-enhanced retention in the mouse brain relative to other organs (70). Moreover, they demonstrated a significant reduction in tumor growth without affecting the weights of the mice. Following this work, Burgess and colleagues compared a meso-Cl substituted gemcitabine-HMCD conjugate (**Figure 3**, **C2**) with **C1 (** 72). The meso-Cl substituted gemcitabine-MHI-148 conjugate was designed with a thiol linker and secondary amide attaching gemcitabine to the dye. This was shown to be readily metabolized into gemcitabine and a modified MHI-148 intermediate within 3 hours, with an *in vivo* half-life of only 1 hour. Hence the instability of the thiol linker and secondary amide bond resulted in cleavage of gemcitabine from the modified MHI-48 intermediate. However, the authors did not show the effect this had on the *in vivo* efficacy of the conjugate. It was also observed that the maximum fluorescent intensity in the liver and tumor was achieved in 30 minutes, and mostly cleared after 24 hours (70). This suggests that meso-Cl displaced conjugates are cleared from the tumor faster than the meso-Cl intact counterparts.

#### Monoamine Oxidase-A Inhibitors: Clorgiline

**Figure 4:**
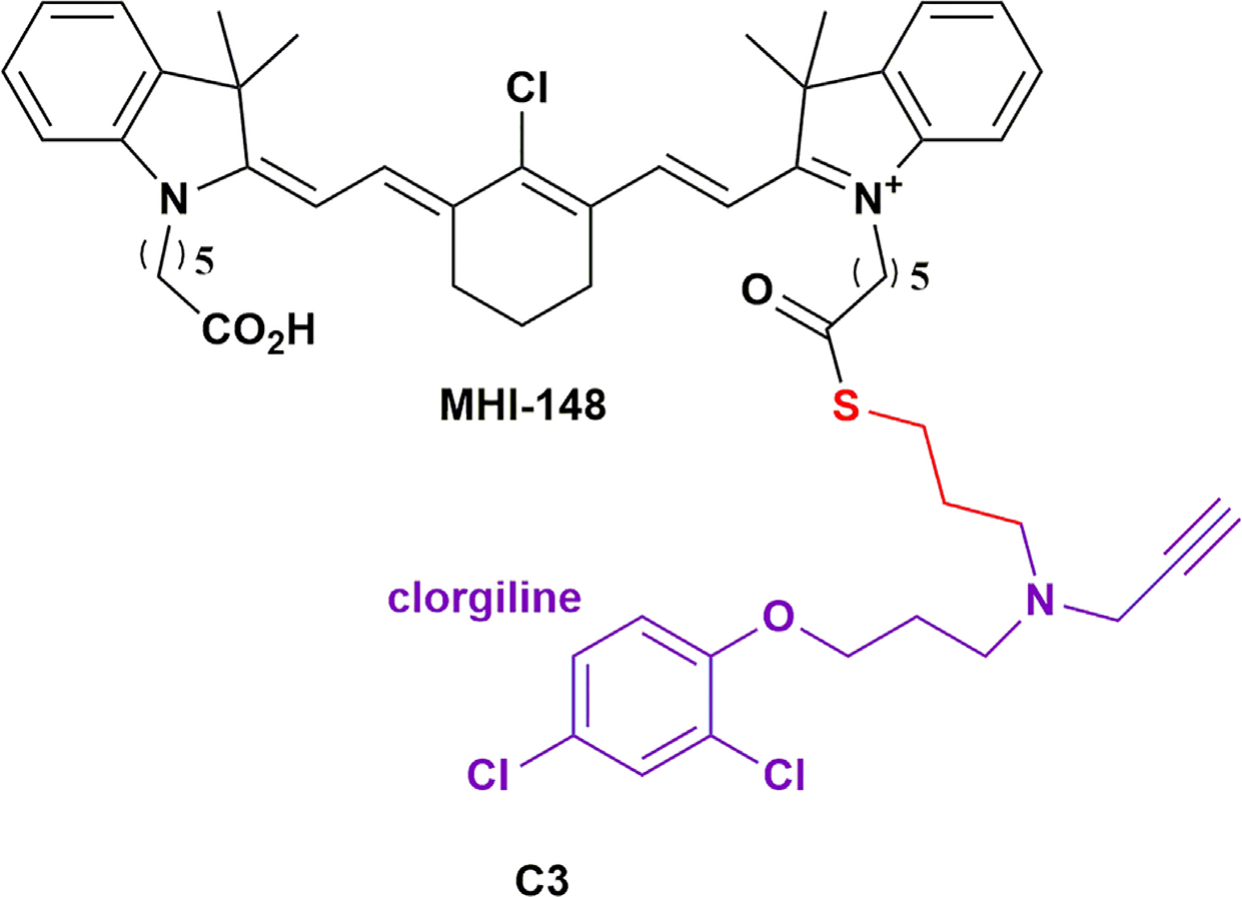
Structure of MAOA inhibitor clorgiline conjugated to MHI-148 (C3) showing which showed tumor specificity in prostate cancer and glioblastoma (114).

Monoamine oxidase-A (MAOA) is a mitochondria-bound enzyme that degrades monoamine neurotransmitters and dietary monoamines (114). MAOA inhibitors are used effectively in the treatment of various neuropsychiatric disorders, but since neurotransmitters are the preferred substrates of MAOA, non-targeted delivery of MAOA for the treatment of cancers would be detrimental (114). Increased MAOA expression has been shown to correlate with prostate cancer progression (65). Wu et al., investigated the efficacy of an MAOA inhibitor HMCD conjugate, **C3** (**Figure 4**; a clorgiline-DZ-1 conjugate), on prostate cancer (65). Evidently, **C3** significantly outperformed clorgiline on inhibiting proliferation and colony formation on three different prostate cancer cell lines. **C3** also significantly inhibited the growth of C4-2B tumor xenografts in mice. Interestingly, intratumoral injection of **C3** into one of two brain tumors within a mouse revealed comparable intensities of the conjugate in both tumors, which is suggestive of rapid redistribution of the injected conjugate between the two tumors. Moreover, intraperitoneal injection of **C3** resulted in selective targeting of both tumors. This highlights the potential of HMCDs as drug delivery carriers for metastatic cancers, and neoplasms in which the bulk of the tumor has been surgically excised. Recently, there is evidence to suggest a role for the MAOA enzyme in the development of GBM in males – the higher risk group for developing the tumor (115). The same group later investigated the delivery of **C3** in glioma, with promising *in vitro* data on glioma cell lines and patient-derived glioma cells (75). They further demonstrated the tumor specificity of **C3** *in vivo*, with no detectable distribution to other organs, leading to increased survival of the mice bearing intracranial tumors from TMZ-resistant human glioma cells (75). Synergism was also observed with low doses of TMZ treatments. The group has a patent pending for MAO inhibitors and their conjugates as therapeutics for the treatment of brain cancer (116).

#### PARPi: Rucaparib

**Figure 5:**
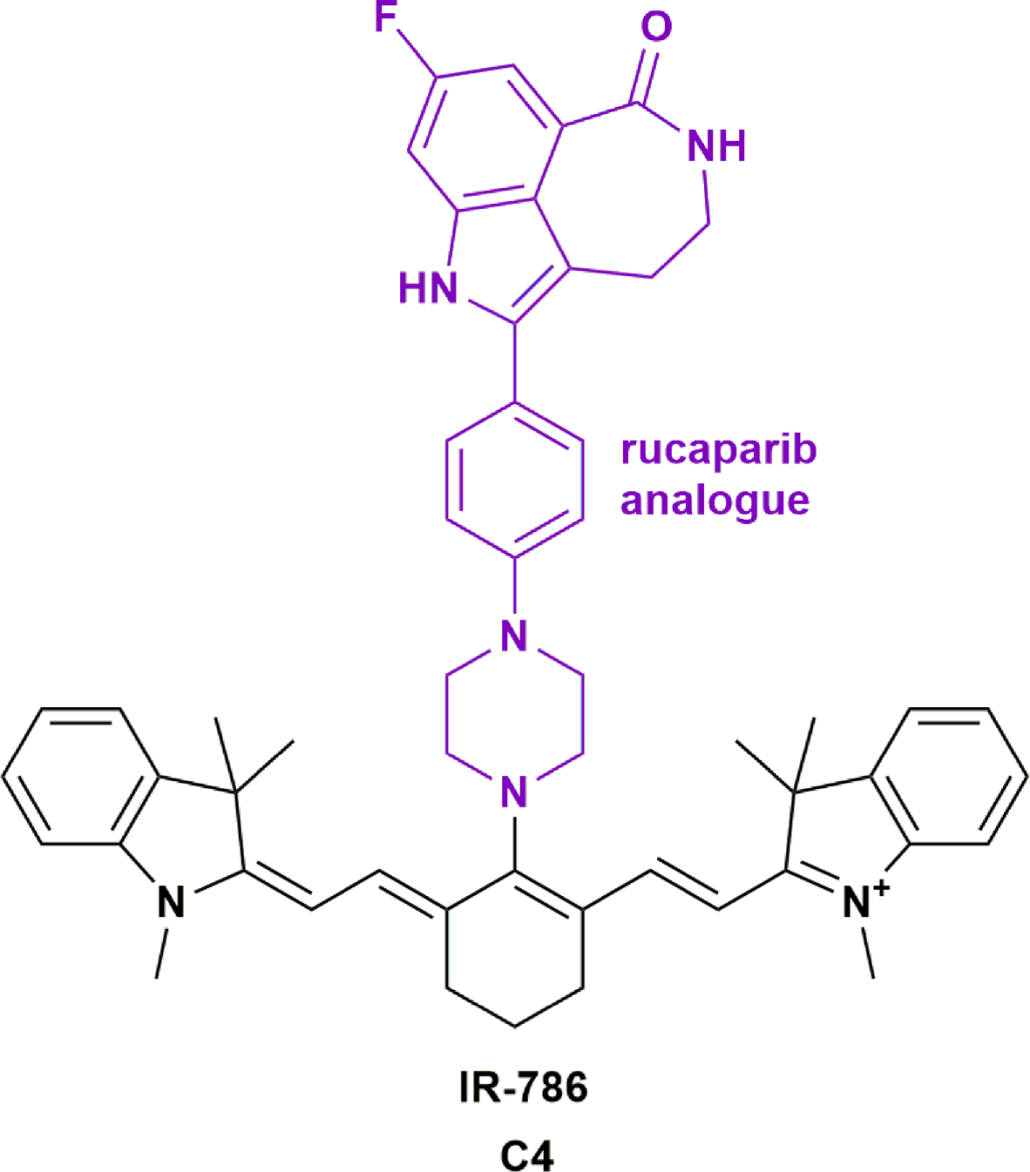
Structure of rucaparib analogue conjugated to IR-786 (C4) showing increased potency in glioblastoma (76).

Poly ADP ribose polymerase (PARP) inhibitors have shown promise in the treatment of many cancers, with several clinical trials underway to expand their indications (NCT04053673, NCT01311713). PARP inhibitors (PARPi) can sensitize glioblastoma cells to radiation, making them a useful adjuvant therapy to use with alkylating agents such as TMZ (117). However, the literature suggests that certain PARPi are substrates for efflux pumps present at the BBB (118), which is thought to be responsible for the limited ability of PARPi to achieve therapeutic concentrations in brain tumors. Moreover, it is reported that PARPi veliparib can radiosensitize normoxic cell lines, which could invoke systemic toxicity (119). Hence, improving the tumor specificity of PARPi could expand their utility for the treatment of brain tumors and metastases. Our group investigated the efficacy of the PARPi rucaparib analogue that was conjugated to IR-786. This resulted in improved potency and specificity on primary patient-derived GBM cell lines when compared to rucaparib alone (**Figure 5**, **C4**) **(** 76). We demonstrated that rucaparib had limited activity across primary patient-derived GBM cell lines. In contrast, conjugation of rucaparib to IR-786 reduced the IC_50_ from 53 μM to 0.02 μM. The conjugate also sensitized GBM cells to TMZ treatment, increasing the toxicity by a further two-fold, despite rucaparib showing no evidence of synergism with TMZ. The radiation-sensitization effects of these compounds are currently being investigated.

### Improving the Therapeutic Potential of Tyrosine Kinase Inhibitors Using HMCDs

Increasing the BBB penetration and specificity of traditional chemotherapy agents are essential in achieving a desirable outcome for patients and reducing neurotoxicity. Recent advances in oncology research have identified aberrant signaling pathways in tumors, which has led to the development of successful TKIs in the treatment of various cancers (17). Despite similar signaling pathways implicated in the tumorigenesis of primary brain tumors and metastases, they have failed to benefit from the recent advances in targeted small molecule inhibitors. In a recent review, Kim and Ko highlighted the number of TKIs that showed negative results in clinical trials, and attributed these failures to the inability of TKIs to cross the BBB (17). Therefore, investigations into overcoming the BBB and increasing the tumor bioavailability would be paramount if TKIs are to be used for the treatment of brain tumors.

TKIs are recognized as powerful candidates for the treatment of GBM, as aberrant tyrosine kinase signaling is central to the pathogenesis. There are two main tyrosine kinase signaling pathways implicated in the tumorigenesis of GBM; the Ras/MEK/ERK pathway, and the PI3K/AKT/mTOR pathways – both of which are being investigated as potential therapeutic targets (120). These complex interconnected pathways originate upstream from common receptor tyrosine kinases, including epidermal growth factor receptors (EGFRs) and PDGFRs (120). Ironically, mutations in these pathways account for 90% of GBM mutations, which include opportunistic deletions of tumor suppressor genes, amplifications and hyper-activation of oncogenes (121). However, clinical trials of TKIs for the treatment of GBM have been unsuccessful, and no TKIs to date have been approved for the treatment of GBM (17).

#### Dasatinib

**Figure 6:**
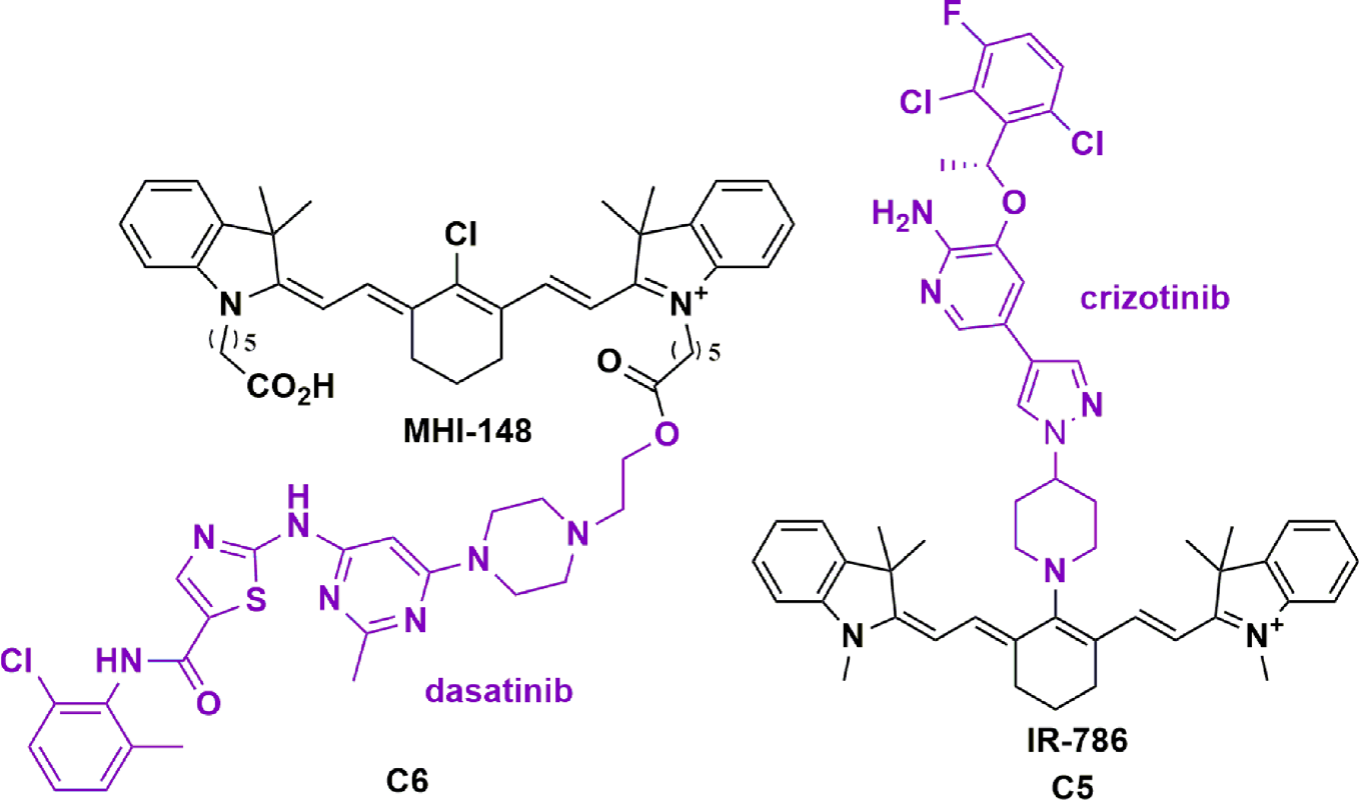
Structure of tyrosine kinase inhibitors dasatinib conjugated to MHI-148 (C5) and crizotinib conjugated to IR-786 (C6) showing tumor specificity and increased potency in glioblastoma (71, 73).

Our group, in addition to Burgess and colleagues, have recently investigated the use of HMCDs to increase the potency and specificity of TKIs in the treatment of GBM (71, 73). Burgess and colleagues have published work on a mono-dasatinib-MHI-148 conjugate in HepG2 cells and the GBM U87 cell line (62, 73). Here, dasatinib was conjugated through an ester coupling to the carboxylic-acid arm of MHI-148 (**Figure 6**, **C5**). The authors highlight a modest 4.7-fold improvement in cell viabilities, despite a 15 and 30-fold reduction in the IC_50_ for Src and Lyn kinases, respectively. Moreover, they demonstrate tumor-specific accumulation of the conjugate for up to 72 hours, with less signals detected in other organs such as the liver and the lungs (122). Interestingly, although MHI-148 has been reported to permeate the BBB in intracranial GBM models, near-infrared images of a healthy mouse brain 4 hours after retro-orbital injection of MHI-148 showed no significant fluorescence, reinforcing the tumor-specificity of HMCDs. The authors suggest that esterase-mediated cleavage of the ester bond in the conjugate could liberate the kinase-inhibitor in a slow-release process, increasing the bioavailability of dasatinib in the tumor parenchyma, but direct evidence is lacking (122).

#### Crizotinib

Similarly, our group has shown that the conjugation of TKI, crizotinib, with HMCD IR-786 through nucleophilic substitution of the meso-Cl can increase the potency of crizotinib by more than 100-fold in primary patient-derived GBM cell lines (**Figure 6**, **C6**) **(** 71). This degree of potency change in relation to the TKI alone was unexpected at an *in vitro* level without the BBB, and further work is required to elucidate its mechanisms. As described, the rationale behind using HMCDs as a drug delivery system is founded on dyes’ brain permeability and tumor specificity. However, the novel aspects of these conjugates could have undiscovered mechanisms that lead to increased potencies in an *in vitro* setting. As alluded to above, more research is required to understand how the drug acts intracellularly, such as whether crizotinib dissociates from the IR-786 compound, or how the conjugate interacts with the target. The crizotinib-IR-786 conjugate worked synergistically with the standard chemotherapy, TMZ to reduce cell viability by a further three-fold (71). At the time of submitting this manuscript, these are the only two TKI-HMCD conjugates reported in the literature, and Burgess and colleagues have a patent pending for the conjugation of kinase inhibitors to HMCDs (123). Given the increased potency of first-generation TKIs conjugated to HMCDs as described, it would be useful to explore the activity of second and third-generation TKIs conjugated to HMCDs both *in vitro* and *in vivo*. For example, second and third generation anaplastic lymphoma kinase inhibitors, brigatinib and lorlatinib, respectively, have also been unsuccessful in the treatment of primary brain tumors, despite achieving intracranial responses in the treatment of metastatic non-small cell lung cancer (124). Overall, the conjugation of TKIs with HMCDs provides a novel approach to overcome the challenges associated with the use of TKIs in the treatment of brain tumors.

## Conclusion and Future Perspectives

Despite the abundance of preclinical trials conducted to identify novel effective therapies for brain tumors and metastases, translation into actual clinical benefits have been limited. *In vivo* and *in vitro* investigations have contributed substantially to our understanding of tumor biology and pathogenesis, providing clear indications for the repurposing of existing anticancer agents. However, the scarcity in recent clinical trials’ success has demonstrated that expanding the indications of existing small molecule anticancer agents for brain tumors and metastases can be challenging. As detailed in this review, the BBB remains a fundamental obstacle for delivering anticancer agents into the brain. Besides, achieving a high degree of tumor specificity and retention, without inflicting additional neurological injury, remains a formidable challenge in obtaining therapeutic effectiveness. For this, HMCDs may provide a unique platform to facilitate the targeted delivery of anticancer agents that have been previously excluded from the brain.

HMCDs are an advantageous candidate for development as a drug-delivery system by virtue of its favourible tumor-specificity and biocompatibility. The primary thing to consider when designing HMCD-drug conjugates is the biological innertness of the carrier system. Literature suggests that the N-alkyl chain length alters the uptake and photophysical properties of HMCDs, but the understanding of the pharmacodynamic and toxicity profiles of different HMCD analogues is lacking (86). A thorough characterization of these properties will identify which groups of HMCDs would serve as the most appropriate drug carrier.

As discussed, the mechanisms of uptake of HMCDs across the BBB and into the tumor cells are unclear. NIRF studies of the biodistribution of HMCDs provides compelling evidence for the tumor-specific accumulation of these dyes and drug conjugates, yet the understanding of the biology of this statement is lacking. The reliance of OATPs for the uptake of HMCDs is generally characterised through pharmacological inhibition of these transporters with a non-specific OATP inhibitor, bromosulfophthalein. However, a more detailed understanding of the expression profile and reliance of these transporters is required. OATPs are ubiquitously expressed throughout the body, hence the biodistribution of HMCDs is an important factor to consider in the development of these compounds.

Particular emphasis should also be laid on the stability of HMCD-drug conjugates. Several groups report an increase in the potency of HMCD-drug conjugates in comparison to the drug alone; however, the mechanism of this is unclear (62, 71, 76). Intracellular unbound drug concentrations determine the affinity to targets within the cell (125). If conjugation of HMCDs with anti-neoplastic agents increases the intracellular drug concentration, it is likely the conjugate will bind to different, or additional drug targets, increasing the potency relative to the unconjugated drug. Measurements of the intracellular accumulation of the HMCD-drug conjugates, in addition to the metabolite profile within tumor cells, will determine whether the increase in potency is related to an increase in the concentration of the drug within the cell, or if the activity is attributed to the formation of a more toxic metabolite.

Other limitations exist in the methodology commonly used to evaluate the activity of HMCD in research. In preclinical studies, the efficacy of HMCD-drug conjugates is generally characterised *in vitro*, and mouse xenograft models are typically used to establish the biodistribution of the HMCD-conjugates in different organs. There is a lack of literature assessing the pharmacodynamic profiles of HMCDs and HMCD-drug conjugates. The *in vitro* accumulation and increased potency of HMCD-conjugates, coupled with the tumor-specific biodistribution is compelling, but few groups have translated this to an increase in overall- or progression-free survival *in vivo.* A thorough analysis of the survival benefits of HMCD-drug conjugates relative to the drug alone in an appropriate *in vivo* model is necessary for the clinical translation of this technology.

Indeed, a deeper insight into deciphering the biological mechanisms of tumor cell uptake and retention of HMCDs are required to enable rational design for their use as drug-carriers. Concomitantly, animal models need to be used more extensively to evaluate their pharmacokinetics and survival benefits. Ultimately, HMCDs provide a novel strategy to reinvigorate existing chemotherapies by exploiting tumor-intrinsic properties to deliver chemotherapies effectively to brain tumors and minimize toxicity.

## Author Contributions

EC: Researched and wrote the entire manuscript. PC: Aided in the writing of the manuscript, especially the medicinal chemistry sections. WD: Supervised the medicinal chemistry sections of the manuscript. JJ: Conceived and supervised the writing of the medicinal chemistry sections of the manuscript. MD: Supervised the biomedical sections of the manuscript. TP: Conceived, wrote, and supervised the entire manuscript. All authors contributed to the article and approved the submitted version.

## Funding

Neurological Foundation of New Zealand; Douglas Charitable Trust; Auckland Cancer Society Research Centre; Hugh Green Foundation.

## Conflict of Interest

The authors declare that the research was conducted in the absence of any commercial or financial relationships that could be construed as a potential conflict of interest.

## Abbreviations

ABC, ATP-binding cassette; BBB, blood brain barrier; CNS, central nervous system; EGFR, epidermal growth factor receptor; EPR, enhanced permeability and retention; GBM, glioblastoma; HSA, human serum albumin; HMCD, heptamethine cyanine dye; MAOA, Monoamine oxidase-A; PDFR, platelet-derived growth factor; NIRF, near infrared dye; OATP, organic-anion transporting polypeptide; PARP, Poly ADP ribose polymerase; TKI; tyrosine kinase inhibitor; TMZ, temozolomide.

## References

[1] Louis DN Perry A Reifenberger G von Deimling A Figarella-Branger D Cavenee WK. The 2016 World Health Organization Classification of Tumors of the Central Nervous System: A Summary. Acta Neuropathol (2016) 131(6):803–20. 10.1007/s00401-016-1545-1 27157931

[2] Miranda-Filho A Piñeros M Soerjomataram I Deltour I Bray F. Cancers of the Brain and CNS: Global Patterns and Trends in Incidence. Neuro Oncol (2017) 19(2):270–80. 10.1093/neuonc/now166 PMC5464292 27571887

[3] Stupp R Mason WP van den Bent MJ Weller M Fisher B Taphoorn MJB. Radiotherapy Plus Concomitant and Adjuvant Temozolomide for Glioblastoma. N Engl J Med (2009) 352(10):987–96. 10.1056/NEJMoa043330 15758009

[4] Jungk C Chatziaslanidou D Ahmadi R Capper D Bermejo JL Exner J. Chemotherapy With BCNU in Recurrent Glioma: Analysis of Clinical Outcome and Side Effects in Chemotherapy-Naïve Patients. BMC Cancer (2016) 16(18):1–11. 10.1186/s12885-016-2131-6 PMC4748520 26865253

[5] Tamura R Tanaka T Miyake K Yoshida K Sasaki H. Bevacizumab for Malignant Gliomas: Current Indications, Mechanisms of Action and Resistance, and Markers of Response. Brain Tumor Pathol (2017) 34(2):62–77. 10.1007/s10014-017-0284-x 28386777

[6] Li Y Ali S Clarke J Cha S. Bevacizumab in Recurrent Glioma: Patterns of Treatment Failure and Implications. Brain Tumor Res Treat (2017) 5(1):1–9. 10.14791/btrt.2017.5.1.1 28516072 PMC5433944

[7] Gan HK Van Den Bent M Lassman AB Reardon DA Scott AM. Antibody-Drug Conjugates in Glioblastoma Therapy: The Right Drugs to the Right Cells. Nat Rev Clin Oncol (2017) 14(11):695–707. 10.1038/nrclinonc.2017.95 28675164

[8] Keating GM. Bevacizumab: A Review of its Use in Advanced Cancer. Drugs (2014) Oct 15 74(16):1891–925. 10.1007/s40265-014-0302-9 25315029

[9] Rosenblum D Joshi N Tao W Karp JM Peer D. Progress and Challenges Towards Targeted Delivery of Cancer Therapeutics. Nat Commun (2018) 9(1):1–12. 10.1038/s41467-018-03705-y 29650952 PMC5897557

[10] Reardon DA Brandes AA Omuro A Mulholland P Lim M Wick A. Effect of Nivolumab vs Bevacizumab in Patients With Recurrent Glioblastoma: The CheckMate 143 Phase 3 Randomized Clinical Trial. JAMA Oncol (2020) 6(7):1003–10. 10.1001/jamaoncol.2020.1024 PMC7243167 32437507

[11] Baker GJ Yadav VN Motsch S Koschmann C Calinescu AA Mineharu Y. Mechanisms of Glioma Formation: Iterative Perivascular Glioma Growth and Invasion Leads to Tumor Progression, VEGF-Independent Vascularization, and Resistance to Antiangiogenic Therapy. Neoplasia (2014) 16(7):543–61. 10.1016/j.neo.2014.06.003 PMC4198934 25117977

[12] Johnston WT Erdmann F Newton R Steliarova-Foucher E Schüz J Roman E. Childhood Cancer: Estimating Regional and Global Incidence. Cancer Epidemiol (2020) 71(4):1877–7821. 10.1016/j.canep.2019.101662 31924557

[13] Khalil J Chaabi S Oberlin O Sialiti S Hessissen L Benjaafar N. Medulloblastoma in Childhood: What Effects on Neurocognitive Functions? Cancer/Radiotherapie (2019) 23(5):370–7. 10.1016/j.canrad.2018.11.004 31331843

[14] Fox BD Cheung VJ Patel AJ Suki D Rao G. Epidemiology of Metastatic Brain Tumors. Neurosurg Clin N Am (2011) 22(1):1–6. 10.1016/j.nec.2010.08.007 21109143

[15] Eichleer AF Chung E Kodack DP Loeffler JS Fukumura D Jain RK. The Biology of Brain Metastases—Translation to New Therapies. Nat Rev Clin Oncol (2012) 8(6):344–56. 10.1038/nrclinonc.2011.58 PMC3259742 21487419

[16] Arvanitis CD Ferraro GB Jain RK. The Blood–Brain Barrier and Blood–Tumour Barrier in Brain Tumours and Metastases. Nat Rev Cancer Nat Research (2020) 20:26–41. 10.1038/s41568-019-0205-x PMC8246629 31601988

[17] Kim G Ko YT. Small Molecule Tyrosine Kinase Inhibitors in Glioblastoma. Arch Pharm Res (2020) 43:385–94. 10.1007/s12272-020-01232-3 32239429

[18] Munoz JL Walker ND Scotto KW Rameshwar P. Temozolomide Competes for P-glycoprotein and Contributes to Chemoresistance in Glioblastoma Cells. Cancer Lett (2015) 367(1):69–75. 10.1016/j.canlet.2015.07.013 26208431

[19] Löscher W Potschka H. Blood-Brain Barrier Active Efflux Transporters: ATP-binding Cassette Gene Family. NeuroRx (2005) 2(1):86–98. 10.1602/neurorx.2.1.86 15717060 PMC539326

[20] Bregy A Shah AH Diaz MV Pierce HE Ames PL Diaz D. The Role of Gliadel Wafers in the Treatment of High-Grade Gliomas. Expert Rev Anticancer Ther (2013) 13(12):1453–61. 10.1586/14737140.2013.840090 24236823

[21] Prados MD Schold SC Fine HA Jaeckle K Hochberg F Mechtler L. A Randomized, Double-Blind, Placebo-Controlled, Phase 2 Study of RMP-7 in Combination With Carboplatin Administered Intravenously for the Treatment of Recurrent Malignant Glioma. Neuro Oncol (2003) 5(2):96–103. 10.1093/neuonc/5.2.96 12672281 PMC1920676

[22] Nam L Coll C Erthal LCS de la Torre C Serrano D Martínez-Máñez R. Drug Delivery Nanosystems for the Localized Treatment of Glioblastoma Multiforme. Mater (Basel Switzerland) (2018) 11(5):779–808. 10.3390/ma11050779 PMC5978156 29751640

[23] Perry J. Gliadel® Wafers in the Treatment of Malignant Glioma: A Systematic Review. Curr Oncol (2007) 14(5):189–94. 10.3747/co.2007.147 PMC2002480 17938702

[24] Chakraborty S Filippi CG Wong T Ray A Fralin S Tsiouris AJ. Superselective Intraarterial Cerebral Infusion of Cetuximab After Osmotic Blood/Brain Barrier Disruption for Recurrent Malignant Glioma: Phase I Study. J Neurooncol (2016) 128(3):405–15. 10.1007/s11060-016-2099-8 26945581

[25] Emerich DF Snodgrass P Dean R Agostino M Hasler B Pink M. Enhanced Delivery of Carboplatin Into Brain Tumours With Intravenous CereportTM (Rmp-7): Dramatic Differences and Insight Gained From Dosing Parameters. Br J Cancer (1999) 80(7):964–70. 10.1038/sj.bjc.6690450 PMC2363031 10362103

[26] Bentourkia M Bol A Ivanoiu A Labar D Sibomana M Coppens A. Comparison of Regional Cerebral Blood Flow and Glucose Metabolism in the Normal Brain: Effect of Aging. J Neurol Sci (2000) 181(1–2):19–28. 10.1016/S0022-510X(00)00396-8 11099707

[27] Nicholson C. Diffusion and Related Transport Mechanisms in Brain Tissue. Rep Prog Phys (2001) 64(7):815. 10.1088/0034-4885/64/7/202

[28] Chaudhry A Benson L Varshaver M Farber O Weinberg U Kirson E. Novottf^tm^-100A System (Tumor Treating Fields) Transducer Array Layout Planning for Glioblastoma: A NovoTAL^TM^ System User Study. World J Surg Oncol (2015) 13(1):316–23. 10.1186/s12957-015-0722-3 PMC4642621 26558989

[29] Stupp R Taillibert S Kanner A Read W Steinberg DM Lhermitte B. Effect of Tumor-Treating Fields Plus Maintenance Temozolomide vs Maintenance Temozolomide Alone on Survival in Patients With Glioblastoma a Randomized Clinical Trial. JAMA - J Am Med Assoc (2017) 318(23):2306–16. 10.1001/jama.2017.18718 PMC5820703 29260225

[30] Beccaria K Canney M Bouchoux G Puget S Grill J Carpentier A. Blood-Brain Barrier Disruption With Low-Intensity Pulsed Ultrasound for the Treatment of Pediatric Brain Tumors: A Review and Perspectives. Neurosurg Focus (2020) 48(1):E10. 10.3171/2019.10.FOCUS19726 31896084

[31] McGregor JM Bell SD Doolittle ND Murillo TP Neuwelt EA. Blood-Brain Barrier Disruption Chemotherapy. In: Newton HB, editor. Handbook of Brain Tumor Chemotherapy, Molecular Therapeutics, and Immunotherapy, 2nd ed. Nikki Levy: Academic Press (2018). p. 145–53.

[32] Wilhelm S Tavares AJ Dai Q Ohta S Audet J Dvorak HF. Analysis of Nanoparticle Delivery to Tumours. Nat Rev Mater (2016) 1(1):1–12. 10.1038/natrevmats.2016.14

[33] Anselmo AC Mitragotri S. Nanoparticles in the Clinic. Bioeng Transl Med (2016) 1(1):10–29. 10.1002/btm2.10003 29313004 PMC5689513

[34] Stone JB DeAngelis LM. Cancer-Treatment-Induced Neurotoxicity-Focus on Newer Treatments. Nat Rev Clin Oncol (2016) 13(2):92–105. 10.1038/nrclinonc.2015.152 26391778 PMC4979320

[35] Raisa N Marhaendraputro EA. The SIDE Effects OF Chemotherapy IN Glioma. MNJ (Malang Neurol Journal) (2019) 5(2):92–7. 10.21776/ub.mnj.2019.005.02.9

[36] Hellström NAK Björk-Eriksson T Blomgren K Kuhn HG. Differential Recovery of Neural Stem Cells in the Subventricular Zone and Dentate Gyrus After Ionizing Radiation. Stem Cells (2009) 27(3):634–41. 10.1634/stemcells.2008-0732 19056908

[37] Matsos A Johnston IN. Chemotherapy-Induced Cognitive Impairments: A Systematic Review of the Animal Literature. Neurosci Biobehav Rev (2019) 102:382–99. 10.1016/j.neubiorev.2019.05.001 31063740

[38] Monje ML Vogel H Masek M Ligon KL Fisher PG Palmer TD. Impaired Human Hippocampal Neurogenesis After Treatment for Central Nervous System Malignancies. Ann Neurol (2007) 62(5):515–20. 10.1002/ana.21214 17786983

[39] Sorrells SF Paredes MF Cebrian-Silla A Sandoval K Qi D Kelley KW. Human Hippocampal Neurogenesis Drops Sharply in Children to Undetectable Levels in Adults. Nature (2018) 555(7696):377–81. 10.1038/nature25975 PMC6179355 29513649

[40] Bagnall-Moreau C Chaudhry S Salas-Ramirez K Ahles T Hubbard K. Chemotherapy-Induced Cognitive Impairment Is Associated With Increased Inflammation and Oxidative Damage in the Hippocampus. Mol Neurobiol (2019) 56(10):7159–72. 10.1007/s12035-019-1589-z PMC6728167 30989632

[41] Matsos A Loomes M Zhou I Macmillan E Sabel I Rotziokos E. Chemotherapy-Induced Cognitive Impairments: White Matter Pathologies. Cancer Treat Rev (2017) 61:6–14. 10.1016/j.ctrv.2017.09.010 29073552

[42] Zikou A Sioka C Alexiou GA Fotopoulos A Voulgaris S Argyropoulou MI. Radiation Necrosis, Pseudoprogression, Pseudoresponse, and Tumor Recurrence: Imaging Challenges for the Evaluation of Treated Gliomas. Contrast Media Mol Imaging (2018) 2018:6828396. 10.1155/2018/6828396 30627060 PMC6305027

[43] Miller JA Bennett EE Xiao R Kotecha R Chao ST Vogelbaum MA. Association Between Radiation Necrosis and Tumor Biology After Stereotactic Radiosurgery for Brain Metastasis. Int J Radiat Oncol Biol Phys (2016) 96(5):1060–9. 10.1016/j.ijrobp.2016.08.039 27742540

[44] Lee ST Seo Y Bae JY Chu K Kim JW Choi SH. Loss of Pericytes in Radiation Necrosis After Glioblastoma Treatments. Mol Neurobiol (2018) 55(6):4918–26. 10.1007/s12035-017-0695-z 28770500

[45] Magge RS DeAngelis LM. The Double-Edged Sword: Neurotoxicity of Chemotherapy. Blood Rev (2015) 29(2):93–100. 10.1016/j.blre.2014.09.012 25445718 PMC5944623

[46] Loprinzi CL Qin R Dakhil SR Fehrenbacher L Flynn KA Atherton P. Phase III Randomized, Placebo-Controlled, Double-Blind Study of Intravenous Calcium and Magnesium to Prevent Oxaliplatin-Induced Sensory Neurotoxicity (N08CB/Alliance). J Clin Oncol (2014) 32(10):997–1005. 10.1200/JCO.2013.52.0536 24297951 PMC3965262

[47] Schloss J Colosimo M Vitetta L. New Insights Into Potential Prevention and Management Options for Chemotherapy-Induced Peripheral Neuropathy. Asia Pacific J Oncol Nurs (2016) 3(1):73. 10.4103/2347-5625.170977 PMC5123533 27981142

[48] Nam L Coll C S Erthal LC de la Torre C Serrano D Martínez-Máñez R. Drug Delivery Nanosystems for the Localized Treatment of Glioblastoma Multiforme. Mater (Basel Switzerland) (2018) 11(5):779–808. 10.3390/ma11050779 PMC5978156 29751640

[49] Guan Y Zhang Y Xiao L Li J Wang J Chordia MD. Improving Therapeutic Potential of Farnesylthiosalicylic Acid: Tumor Specific Delivery Via Conjugation With Heptamethine Cyanine Dye. Mol Pharm (2017) 14(1):1–13. 10.1021/acs.molpharmaceut.5b00906 26992462 PMC5815365

[50] Wang Y Liu T Zhang E Luo S Tan X Biomaterials C--. Preferential Accumulation of the Near Infrared Heptamethine Dye IR-780 in the Mitochondria of Drug-Resistant Lung Cancer Cells. Biomaterials (2014) 35(13):4116–24. 10.1016/j.biomaterials.2014.01.061 24529902

[51] Luo S Tan X Qi Q Guo Q Ran X Zhang L. A Multifunctional Heptamethine Near-Infrared Dye for Cancer Theranosis. Biomaterials (2013) 34(9):2244–51. 10.1016/j.biomaterials.2012.11.057 23261220

[52] Tan X Luo S Wang D Su Y Cheng T Shi C. A NIR Heptamethine Dye With Intrinsic Cancer Targeting, Imaging and Photosensitizing Properties. Biomaterials (2012) 33(7):2230–9. 10.1016/j.biomaterials.2011.11.081 22182749

[53] Yang X Shi C Tong R Qian W Zhau HE Wang R. Near IR Heptamethine Cyanine Dye-Mediated Cancer Imaging. Clin Cancer Res (2010) 16(10):2833–44. 10.1158/1078-0432.CCR-10-0059 PMC2871283 20410058

[54] Zhang E Luo S Tan X Biomaterials C--. 2014 U. Mechanistic Study of IR-780 Dye as a Potential Tumor Targeting and Drug Delivery Agent. Biomaterials (2014) 35(2):771–8. 10.1016/j.biomaterials.2013.10.033 24148240

[55] Li S Sun Z Meng X Deng G Zhang J Zhou K. Targeted Methotrexate Prodrug Conjugated With Heptamethine Cyanine Dye Improving Chemotherapy and Monitoring Itself Activating by Dual-Modal Imaging. Front Mater (2018) 5:35. 10.3389/fmats.2018.00035

[56] Zhang C Long L Shi C. Mitochondria-Targeting IR-780 Dye and Its Derivatives: Synthesis, Mechanisms of Action, and Theranostic Applications. Adv Ther (2018) 1(7):1800069. 10.1002/adtp.201800069

[57] Yang X Hou Z Wang D Mou Y Guo C. Design, Synthesis and Biological Evaluation of Novel Heptamethine Cyanine Dye-Erlotinib Conjugates as Antitumor Agents. Bioorganic Med Chem Lett (2020) 30(23):127557. 10.1016/j.bmcl.2020.127557 32949719

[58] Guan Y Zhang Y Zou J Huang LP Chordia MD Yue W. Synthesis and Biological Evaluation of genistein-IR783 Conjugate: Cancer Cell Targeted Delivery in MCF-7 for Superior Anti-Cancer Therapy. Molecules (2019) 24(22):2–17. 10.3390/molecules24224120 PMC6891397 31739548

[59] Harrison VSR Carney CE MacRenaris KW Waters EA Meade TJ. Multimeric Near Ir-Mr Contrast Agent for Multimodal in Vivo Imaging. J Am Chem Soc (2015) 137(28):9108–16. 10.1021/jacs.5b04509 PMC4512902 26083313

[60] Zhang C Liu T Su Y Luo S Zhu Y Tan X. A Near-Infrared Fluorescent Heptamethine Indocyanine Dye With Preferential Tumor Accumulation for In Vivo Imaging. Biomaterials (2010) 31(25):6612–7. 10.1016/j.biomaterials.2010.05.007 20542559

[61] Li P Liu Y Liu W Li G Tang Q Zhang Q. Ir-783 Inhibits Breast Cancer Cell Proliferation and Migration by Inducing Mitochondrial Fission. Int J Oncol (2019) 55(2):415–24. 10.3892/ijo.2019.4821/abstract PMC6615916 31173174

[62] Usama SM Zhao B Burgess K. A Near-IR Fluorescent Dasatinib Derivative That Localizes in Cancer Cells. Bioconjug Chem (2019) Apr 17 30(4):1175–81. 10.1021/acs.bioconjchem.9b00118 PMC7050276 30931563

[63] Wu J Shao C Li X Shi C Li Q Hu P. Near-Infrared Fluorescence Imaging of Cancer Mediated by Tumor Hypoxia and HIF1α/Oatps Signaling Axis. Biomaterials (2014) 35(28):8175–85. 10.1016/j.biomaterials.2014.05.073 PMC4180424 24957295

[64] Yi X Yan F Wang F Qin W Wu G. Ir-780 Dye for Near-Infrared Fluorescence Imaging in Prostate Cancer. Med Sci Monit (2015) 21:511–7. 10.12659/MSM.892437 PMC4335586 25686161

[65] Wu JB Lin T Gallagher JD Kushal S Chung LWK Zhau HE. Monoamine Oxidase A Inhibitor – Near-Infrared Dye Conjugate Reduces Prostate Tumor Growth. J Am Chem Soc (2015) 137(6):2366–74. 10.1021/ja512613j 25585152

[66] Shi C Wu J Chu G Li Q Wang R Oncotarget C--. Heptamethine Carbocyanine Dye-Mediated Near-Infrared Imaging of Canine and Human Cancers Through the HIF-1α/Oatps Signaling Axis. Oncota (2014) 5:10114–26. 10.18632/oncotarget.2464 PMC4259409 25361418

[67] Lv Q Wang D Yang Z Yang J Zhang R Yang X. Repurposing Antitubercular Agent Isoniazid for Treatment of Prostate Cancer. Biomater Sci (2019) 7(1):296–306. 10.1039/c8bm01189c 30468220

[68] Ma X Laramie M Henary M. Synthesis, Optical Properties and Cytotoxicity of Meso-Heteroatom Substituted IR-786 Analogs. Bioorganic Med Chem Lett (2018) 28(3):509–14. 10.1016/j.bmcl.2017.12.001 29249562

[69] Yang X Shao C Wang R Chu CY Hu P Master V. Optical Imaging of Kidney Cancer With Novel Near Infrared Heptamethine Carbocyanine Fluorescent Dyes. J Urol (2013) 189(2):702–10. 10.1016/j.juro.2012.09.056 PMC4120709 23000848

[70] Boyang J Shi C Chu GC Xu Q Zhang Y Li Q. Biomaterials Near-infrared Fl Uorescence Heptamethine Carbocyanine Dyes Mediate Imaging and Targeted Drug Delivery for Human Brain Tumor. Biomaterials (2015) 67:1–10. 10.1016/j.biomaterials.2015.07.028 26197410 PMC4736505

[71] Choi PJ Cooper E Schweder P Mee E Faull R Denny WA. The Synthesis of a Novel Crizotinib Heptamethine Cyanine Dye Conjugate That Potentiates the Cytostatic and Cytotoxic Effects of Crizotinib in Patient-Derived Glioblastoma Cell Lines. Bioorg Med Chem Lett (2019) 29(18):2617–21. 10.1016/j.bmcl.2019.07.051 31378572

[72] Jiang Z Pflug K Usama SM Kuai D Yan X Sitcheran R. Cyanine-Gemcitabine Conjugates as Targeted Theranostic Agents for Glioblastoma Tumor Cells. J Med Chem (2019) 62(20):9236–45. 10.1021/acs.jmedchem.9b01147 PMC7050787 31469566

[73] Usama SM Jiang Z Pflug K Sitcheran R Burgess K. Conjugation of Dasatinib With MHI-148 has a Significant Advantageous Effect in Viability Assays for Glioblastoma. Chem Med Chem Commun (2019) 14(17):1575–9. 10.1002/cmdc.201900356 31322832

[74] Usama SM Lin C-M Burgess K. On the Mechanisms of Uptake of Tumor-Seeking Cyanine Dyes. Bioconjug Chem (2018) 29(11):3886–95. 10.1021/acs.bioconjchem.8b00708 30354072

[75] Kushal S Wang W Vaikari VP Kota R Chen TC Hofman FM. Monoamine Oxidase A ( Mao A ) Inhibitors Decrease Glioma Progression. Oncotarget (2016) 7(12):13842–53. 10.18632/oncotarget.7283 PMC4924682 26871599

[76] Choi PJ Cooper E Schweder P Mee E Turner C Faull R. PARP Inhibitor Cyanine Dye Conjugate With Enhanced Cytotoxic and Antiproliferative Activity in Patient Derived Glioblastoma Cell Lines. Bioorg Med Chem Lett (2020) 30(14):127252. 10.1016/j.bmcl.2020.127252 32527552

[77] Zhang C Liu T Su Y Luo S Zhu Y Tan X. A Near-Infrared Fluorescent Heptamethine Indocyanine Dye With Preferential Tumor Accumulation for In Vivo Imaging. Biomaterials (2010) 31(25):6612–7. 10.1016/j.biomaterials.2010.05.007 20542559

[78] Choi PJ Park H Cooper E Dragunow M Denny WA Jose J. Heptamethine Cyanine Dye Mediated Drug Delivery: Hype or Hope. Bioconjug Chem (2020) 31(7):1724–39. 10.1021/acs.bioconjchem.0c00302 32530288

[79] Shi C Wu JB Pan D. Review on Near-Infrared Heptamethine Cyanine Dyes as Theranostic Agents for Tumor Imaging, Targeting, and Photodynamic Therapy. J BioMed Opt (2016) 21(5):50901. 10.1117/1.JBO.21.5.050901 27165449

[80] Watson JR Martirosyan N Lemole GM Trouard TP Romanowski M. Intraoperative Brain Tumor Resection With Indocyanine Green Using Augmented Microscopy. J BioMed Opt (2018) 23(09):1. 10.1117/1.JBO.23.9.090501.full PMC6170140 30251491

[81] Ginimuge PR Prashant R Jyothi SD. Methylene Blue: Revisited. J Anaesthesiol Clin Pharmacol (2010) 26(4):517–20. PMC3087269 21547182

[82] Traylor JI Pernik MN Sternisha AC McBrayer SK Abdullah KG. Molecular and Metabolic Mechanisms Underlying Selective 5-Aminolevulinic Acid-Induced Fluorescence in Gliomas. Cancers (Basel) (2021) 13(3):1–15. 10.3390/cancers13030580 PMC7867275 33540759

[83] Charalampaki P Proskynitopoulos PJ Heimann A Nakamura M. 5-Aminolevulinic Acid Multispectral Imaging for the Fluorescence-Guided Resection of Brain Tumors: A Prospective Observational Study. Front Oncol (2020) 10:1069–79. 10.3389/fonc.2020.01069 32733798 PMC7362891

[84] Wu J Shi C Chu G Xu Q Zhang Y Li Q. Near-Infrared Fluorescence Heptamethine Carbocyanine Dyes Mediate Imaging and Targeted Drug Delivery for Human Brain Tumor. Biomaterials (2015) 67:1–10. 10.1016/j.biomaterials.2015.07.028 26197410 PMC4736505

[85] Shao C Liao CP Hu P Chu CY Zhang L Bui MHT. Detection of Live Circulating Tumor Cells by a Class of Near-Infrared Heptamethine Carbocyanine Dyes in Patients With Localized and Metastatic Prostate Cancer. PloS One (2014) 9(2):1–11. 10.1371/journal.pone.0088967 PMC3925210 24551200

[86] Yadav Y Levitz A Dharma S Aneja R Henary M. Effects of Heterocyclic N-alkyl Chain Length on Cancer Cell Uptake of Near Infrared Heptamethine Cyanine Dyes. Dye Pigment (2017) 145:307–14. 10.1016/j.dyepig.2017.06.016

[87] Li Y Zhou Q Deng Z Pan M Liu X Wu J. Ir-780 Dye as a Sonosensitizer for Sonodynamic Therapy of Breast Tumor. Sci Rep (2016) 6(1):1–10. 10.1038/srep25968 27174006 PMC4865802

[88] Levêque D Becker G Bilger K Natarajan-Amé S. Clinical Pharmacokinetics and Pharmacodynamics of Dasatinib. Clin Pharmacokinet (2020) 59(7):849–56. 10.1007/s40262-020-00872-4 32112275

[89] Mittapalli RK Chung AH Parrish KE Crabtree D Halvorson KG Hu G. ABCG2 and ABCB1 Limit the Efficacy of Dasatinib in a PDGF-B-Driven Brainstem Glioma Model. Mol Cancer Ther (2016) 15(5):819–29. 10.1158/1535-7163.MCT-15-0093 PMC4873451 26883271

[90] Lassman AB Pugh SL Gilbert MR Aldape KD Geinoz S Beumer JH. Phase 2 Trial of Dasatinib in Target-Selected Patients With Recurrent Glioblastoma (RTOG 0627). Neuro Oncol (2015) 17(7):992–8. 10.1093/neuonc/nov011 PMC5762006 25758746

[91] Galanis E Anderson SK Twohy EL Carrero XW Dixon JG Tran DD. A Phase 1 and Randomized, Placebo-Controlled Phase 2 Trial of Bevacizumab Plus Dasatinib in Patients With Recurrent Glioblastoma: Alliance/North Central Cancer Treatment Group N0872. Cancer (2019) 125(21):3790–800. 10.1002/cncr.32340 PMC6788934 31290996

[92] Obaidat A Roth M Hagenbuch B. The Expression and Function of Organic Anion Transporting Polypeptides in Normal Tissues and in Cancer. Annu Rev Pharmacol Toxicol (2012) 52(1):135–51. 10.1146/annurev-pharmtox-010510-100556 PMC3257355 21854228

[93] Tamai I Nakanishi T. OATP Transporter-Mediated Drug Absorption and Interaction. Curr Opin Pharmacol (2013) 13(6):859–63. 10.1016/j.coph.2013.09.001 24060700

[94] Wang Y Liao X Sun J Yi B Luo S Liu T. Characterization of HIF-1α/Glycolysis Hyperactive Cell Population Via Small-Molecule-Based Imaging of Mitochondrial Transporter Activity. Adv Sci (2018) 5(3):1700392. 10.1002/advs.201700392 PMC5867035 29593950

[95] Canovas C Bellaye PS Moreau M Romieu A Denat F Goncalves V. Site-Specific Near-Infrared Fluorescent Labelling of Proteins on Cysteine Residues With: Meso -Chloro-Substituted Heptamethine Cyanine Dyes. Org Biomol Chem (2018) 16(45):8831–6. 10.1039/C8OB02646G 30411777

[96] Lin CM Usama SM Burgess K. Site-Specific Labeling of Proteins With near-IR Heptamethine Cyanine Dyes. Molecules (2018) 23(11):1–11. 10.3390/molecules23112900 PMC6278338 30405016

[97] JO J Song MG Park JY Youn H Chung J-K Jeong JM. Fluorescence Labeled Human Serum Albumin as an Imaging Agent for a SPARC(secreted Protein Acidic and Rich in Cysteine) Expressing Glioblastoma. J Nucl Med (2017) 58(supplement 1):53–3. 10.7150/thno.34883

[98] Heneweer C Holland JP Divilov V Carlin S Lewis JS. Magnitude of Enhanced Permeability and Retention Effect in Tumors With Different Phenotypes: 89Zr-Albumin as a Model System. J Nucl Med (2011) 52(4):625–33. 10.2967/jnumed.110.083998 PMC3902086 21421727

[99] Kalyane D Raval N Maheshwari R Tambe V Kalia K Tekade RK. Employment of Enhanced Permeability and Retention Effect (EPR): Nanoparticle-based Precision Tools for Targeting of Therapeutic and Diagnostic Agent in Cancer. Mater Sci Eng C (2019) 98:1252–76. 10.1016/j.msec.2019.01.066 30813007

[100] Von Hoff DD Ervin T Arena FP Chiorean EG Infante J Moore M. Increased Survival in Pancreatic Cancer With Nab-Paclitaxel Plus Gemcitabine 2013. N Engl J Med (2013) 369(18):1691–703. 10.1056/NEJMoa1304369 PMC4631139 24131140

[101] Green MR Manikhas GM Orlov S Afanasyev B Makhson AM Bhar P. Abraxane, a Novel Cremophor-free, Albumin-Bound Particle Form of Paclitaxel for the Treatment of Advanced non-Small-Cell Lung Cancer. Ann Oncol Off J Eur Soc Med Oncol (2006) 17(8):1263–8. 10.1093/annonc/mdl104 16740598

[102] Desai N Trieu V Yao Z Louie L Ci S Yang A. Increased Antitumor Activity, Intratumor Paclitaxel Concentrations, and Endothelial Cell Transport of Cremophor-Free, Albumin-Bound Paclitaxel, ABI-007, Compared With Cremophor-Based Paclitaxel. Clin Cancer Res (2006) 12(4):1317–24. 10.1158/1078-0432.CCR-05-1634 16489089

[103] Cho SS Salinas R Lee JYK. Indocyanine-Green for Fluorescence-Guided Surgery of Brain Tumors: Evidence, Techniques, and Practical Experience. Front Surg (2019) 6:11/full. 10.3389/fsurg.2019.00011/full 30915339 PMC6422908

[104] Larsen MT Kuhlmann M Hvam ML Howard KA. Albumin-Based Drug Delivery: Harnessing Nature to Cure Disease. Mol Cell Ther (2016) 4(1):1–12. 10.1186/s40591-016-0048-8 26925240 PMC4769556

[105] Narazaki R Hamada M Harada K Otagiri M. Covalent Binding Between Bucillamine Derivatives and Human Serum Albumin. Pharm Res (1996) 13(9):1317–21. 10.1023/A:1016057513490 8893268

[106] Frangioni JV Onishi S. Serum Albumin Conjugated to Fluorescent Substances for Imaging. United States of America: World Intellectual Property Organisation. US20080308744A1 (2005) p. 1–7. Available at: https://patents.google.com/patent/WO2005082423A3/en.

[107] Awasthi K Nishimura G. Modification of Near-Infrared Cyanine Dyes by Serum Albumin Protein. Photochem Photobiol Sci (2011) 10(4):461–3. 10.1039/c0pp00271b 21152615

[108] Usama SM Park GK Nomura S Baek Y Choi HS Burgess K. Role of Albumin in Accumulation and Persistence of Tumor-Seeking Cyanine Dyes. Bioconjug Chem (2020) 31(2):248–59. 10.1021/acs.bioconjchem.9b00771 PMC7174984 31909595

[109] Frei E. Albumin Binding Ligands and Albumin Conjugate Uptake by Cancer Cells. Diabetol Metab Syndr (2011) 3(1):11. 10.1186/1758-5996-3-11 21676260 PMC3133998

[110] Chatterjee M Ben-Josef E Robb R Vedaie M Seum S Thirumoorthy K. Caveolae-Mediated Endocytosis is Critical for Albumin Cellular Uptake and Response to Albumin-Bound Chemotherapy. Cancer Res (2017) 77(21):5925–37. 10.1158/0008-5472.CAN-17-0604 PMC5668166 28923854

[111] Lin T Zhao P Jiang Y Tang Y Jin H Pan Z. Blood-Brain-Barrier-Penetrating Albumin Nanoparticles for Biomimetic Drug Delivery Via Albumin-Binding Protein Pathways for Antiglioma Therapy. ACS Nano (2016) 10(11):9999–10012. 10.1021/acsnano.6b04268 27934069

[112] Akinc A Battaglia G. Exploiting Endocytosis for Nanomedicines. Cold Spring Harb Perspect Biol (2013) 5(11):a016980. 10.1101/cshperspect.a016980 24186069 PMC3809578

[113] Bergman AM Pinedo HM Peters GJ. Determinants of Resistance to 2 ,2-Difluorodeoxycytidine (Gemcitabine). Drug Resist Update (2002) 5(1):19–33. 10.1016/s1368-7646(02)00002-x 12127861

[114] Youdim MBH Edmondson D Tipton KF. The Therapeutic Potential of Monoamine Oxidase Inhibitors. Nat Rev Neurosci (2006) 7(4):295–309. 10.1038/nrn1883 16552415

[115] Sjöberg RL Wu WYY Dahlin AM Tsavachidis S Bondy ML Melin B. Role of Monoamine-Oxidase-A-Gene Variation in the Development of Glioblastoma in Males: A Case Control Study. J Neurooncol (2019) 145(2):287–94. 10.1007/s11060-019-03294-w PMC6856259 31556016

[116] Shih JC Hofman FM Chen TC. Mao Inhibitors and Their Conjugates as Therapeutics for the Treatment of Brain Cancer. United States of America: World Intellectual Property Organization; Wo 2015/120206 A1. (2015).

[117] Lesueur P Lequesne J Grellard JM Dugué A Coquan E Brachet PE. Phase I/IIa Study of Concomitant Radiotherapy With Olaparib and Temozolomide in Unresectable or Partially Resectable Glioblastoma: OLA-TMZ-RTE-01 Trial Protocol. BMC Cancer (2019) 19(1):198. 10.1186/s12885-019-5413-y 30832617 PMC6399862

[118] Kizilbash SH Gupta SK Chang K Kawashima R Parrish KE Carlson BL. Restricted Delivery of Talazoparib Across the Blood–Brain Barrier Limits the Sensitizing Effects of PARP Inhibition on Temozolomide Therapy in Glioblastoma. Mol Cancer Ther (2017) 16(12):2735–46. 10.1158/1535-7163.MCT-17-0365 PMC5716902 28947502

[119] Liu SK Coackley C Krause M Jalali F Chan N Bristow RG. A Novel Poly(ADP-Ribose) Polymerase Inhibitor, ABT-888, Radiosensitizes Malignant Human Cell Lines Under Hypoxia. Radiother Oncol (2008) 88(2):258–68. 10.1016/j.radonc.2008.04.005 18456354

[120] Nakada M Kita D Watanabe T Hayashi Y Teng L Pyko IV. Aberrant Signaling Pathways in Glioma. Cancers (Basel) (2011) 3(3):3242–78. 10.3390/cancers3033242 PMC3759196 24212955

[121] McLendon R Friedman A Bigner D Van Meir EG Brat DJ Mastrogianakis GM. Comprehensive Genomic Characterization Defines Human Glioblastoma Genes and Core Pathways. Nature (2008) 455:1061–8. 10.1038/nature07385 PMC2671642 18772890

[122] Usama SM Zhao B Burgess K. A Near-IR Fluorescent Dasatinib Derivative That Localizes in Cancer Cells. Bioconjug Chem (2019) 30(4):1175–81. 10.1021/acs.bioconjchem.9b00118 PMC7050276 30931563

[123] Usama SM Burgess K. Conjugates of Kinase Inhibitors and Cyanine Dyes. United States of America; US 2019/0343958 A1 (2019).

[124] Ross Camidge D Kim DW Tiseo M Langer CJ Ahn MJ Shaw AT. Exploratory Analysis of Brigatinib Activity in Patients With Anaplastic Lymphoma Kinase-Positive non–Small-Cell Lung Cancer and Brain Metastases in Two Clinical Trials. J Clin Oncol (2018) 36(26):2693–701. 10.1200/JCO.2017.77.5841 29768119

[125] Mateus A Matsson P Artursson P. Rapid Measurement of Intracellular Unbound Drug Concentrations. Mol Pharm (2013) 10(6):2467–78. 10.1021/mp4000822 23631740

